# Autism and Schizophrenia-Associated CYFIP1 Regulates the Balance of Synaptic Excitation and Inhibition

**DOI:** 10.1016/j.celrep.2019.01.092

**Published:** 2019-02-19

**Authors:** Elizabeth C. Davenport, Blanka R. Szulc, James Drew, James Taylor, Toby Morgan, Nathalie F. Higgs, Guillermo López-Doménech, Josef T. Kittler

**Affiliations:** 1 Department of Neuroscience, Physiology and Pharmacology, University College London, Gower Street, London WC1E 6BT, UK

**Keywords:** excitatory/inhibitory, CNV, ASD, GABA, dendritic spine, gephyrin, epilepsy, 15q11.2, microduplication, microdeletion

## Abstract

Altered excitatory/inhibitory (E/I) balance is implicated in neuropsychiatric and neurodevelopmental disorders, but the underlying genetic etiology remains poorly understood. Copy number variations in CYFIP1 are associated with autism, schizophrenia, and intellectual disability, but its role in regulating synaptic inhibition or E/I balance remains unclear. We show that CYFIP1, and the paralog CYFIP2, are enriched at inhibitory postsynaptic sites. While CYFIP1 or CYFIP2 upregulation increases excitatory synapse number and the frequency of miniature excitatory postsynaptic currents (mEPSCs), it has the opposite effect at inhibitory synapses, decreasing their size and the amplitude of miniature inhibitory postsynaptic currents (mIPSCs). Contrary to CYFIP1 upregulation, its loss *in vivo*, upon conditional knockout in neocortical principal cells, increases expression of postsynaptic GABA_A_ receptor β2/3-subunits and neuroligin 3, enhancing synaptic inhibition. Thus, CYFIP1 dosage can bi-directionally impact inhibitory synaptic structure and function, potentially leading to altered E/I balance and circuit dysfunction in CYFIP1-associated neurological disorders.

## Highlights

• CYFIP1 and CYFIP2 are enriched at inhibitory synapses.
• CYFIP1 upregulation differentially disrupts inhibitory and excitatory synapses.
• Conditional loss of CYFIP1 alters neuroligin 3 and GABA_A_R β-subunits expression.
• Loss of CYFIP1 increases inhibitory synaptic clusters and hence mIPSC amplitude.

## Graphical Abstract

**Figure:**
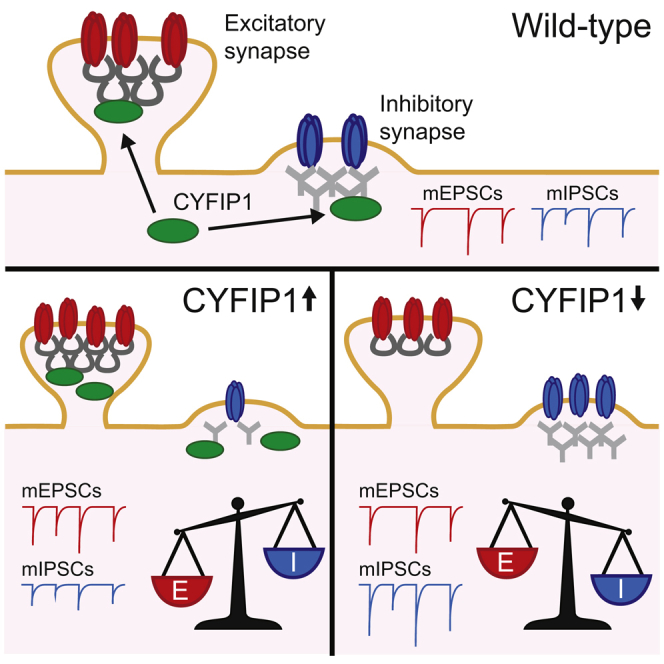

## Teaser

Copy number variations (CNVs) in *CYFIP1* are associated with neuropsychiatric disorders. Davenport et al. uncover an essential role for CYFIP1 in regulating synaptic inhibition and in maintaining the excitatory/inhibitory balance. By altering CYFIP1 gene dosage, to model CNV, they reveal important bi-directional effects on inhibitory transmission in hippocampal neurons.

## Introduction

Schizophrenia (SCZ) and autism spectrum disorder (ASD) have a strong genetic component with a growing number of rare variant mutations and copy number variations (CNVs) (deletions and duplications) in functionally overlapping synaptic and neurodevelopmental gene sets linked to increased disease susceptibility (Bourgeron, 2015, Fromer et al., 2014, Iossifov et al., 2014, Marshall et al., 2017, De Rubeis et al., 2014). Identifying how neuronal connectivity is altered by these genetic lesions is crucial for understanding nervous system function and pathology. CNVs of the 15q11.2 region of the human genome are implicated in the development of neurological and neuropsychiatric conditions. 15q11.2 CNV loss is associated with SCZ (Marshall et al., 2017, Rees et al., 2014, Stefansson et al., 2008), while numerous reports have identified 15q11.2 duplications and deletions in individuals with ASD (Doornbos et al., 2009, Picinelli et al., 2016, Pinto et al., 2014, van der Zwaag et al., 2010), epilepsy, and intellectual disability (de Kovel et al., 2010, Nebel et al., 2016, Vanlerberghe et al., 2015). 15q11.2 contains four genes (NIPA1, NIPA2, CYFIP1, and TUBGCP5) with substantial evidence from rodent and human models pointing toward CYFIP1 as the key disease-causing gene (Bozdagi et al., 2012, Nebel et al., 2016, Oguro-Ando et al., 2015, Pathania et al., 2014, De Rubeis et al., 2013, Yoon et al., 2014). Polymorphisms and rare variants in CYFIP1 are also linked to susceptibility in ASD (Toma et al., 2014, Wang et al., 2015) and SCZ (Tam et al., 2010, Yoon et al., 2014) with a direct deletion of *CYFIP1* identified in an autistic patient with a *SHANK2* deletion (Leblond et al., 2012). Moreover, an upregulation of CYFIP1 mRNA has been observed in ASD patients with a duplication in 15q11-13, highlighting the importance of investigating the effects of genetic duplication as well as deletion (Nishimura et al., 2007, Oguro-Ando et al., 2015). The CYFIP1 paralog, CYFIP2, has also been linked to neurological disorders including SCZ, epilepsy, eating disorders, Alzheimer’s disease, fragile X syndrome-like behaviors, and cocaine seeking (Föcking et al., 2015, Han et al., 2015, Kirkpatrick et al., 2017, Kumar et al., 2013, Nakashima et al., 2018, Tiwari et al., 2016).

CYFIP1 and CYFIP2 are key components of the WAVE regulatory complex (WRC) (a hetero-pentamer consisting of WAVE, Abi, Nap1, HSPC300, and CYFIP1 or CYFIP2) that plays a critical role in regulating the dynamics of the actin cytoskeleton in cells by activating ARP2/3-mediated F-actin branching (Chen et al., 2010). Rare variants of Nap1 (NCKAP1) are also genetically linked to ASD and intellectual disability (Anazi et al., 2017, Iossifov et al., 2014, De Rubeis et al., 2014), providing further genetic support for a critical role of WRC-dependent actin regulatory pathways in neurodevelopmental disorders. Additionally, CYFIP1 is also a repressor of cap-dependent translation by acting as a non-canonical eIF4E binding protein in its complex with the ASD-associated FMRP protein (Napoli et al., 2008) and can also modulate the mTOR pathway (Oguro-Ando et al., 2015).

Synaptic inhibition, mediated by GABA_A_ receptors (GABA_A_Rs), is vital for the efficient control of network excitability, excitation/inhibition (E/I) balance, and for normal brain function. Inhibitory synapses require the stabilization of postsynaptic GABA_A_Rs opposed to GABA-releasing presynaptic terminals. Modulation of inhibitory synaptic strength can be achieved by regulating the size and number of inhibitory synapses (Bannai et al., 2009, Muir et al., 2010, Twelvetrees et al., 2010) and the clustering of GABA_A_Rs by an inhibitory postsynaptic complex containing the gephyrin scaffold (Tyagarajan and Fritschy, 2014), in addition to membrane proteins and adhesion molecules such as LHFPL4 and neuroligins (Davenport et al., 2017, Pettem et al., 2013, Poulopoulos et al., 2009, Smith et al., 2014, Uezu et al., 2016, Yamasaki et al., 2017). While CYFIP1 is enriched at excitatory synapses where it can regulate F-actin dynamics (Pathania et al., 2014) and the development and plasticity of dendritic spines (Abekhoukh et al., 2017, Pathania et al., 2014, De Rubeis et al., 2013), the role of CYFIP1 at inhibitory synapses and in regulating the E/I balance remains undetermined.

Here, we show that CYFIP1 and CYFIP2 are enriched at inhibitory synapses. CYFIP1 upregulation in dissociated neurons, to model microduplication, alters the excitatory-to-inhibitory synapse ratio, resulting in reduced miniature inhibitory postsynaptic current (mIPSC) amplitude and increased miniature excitatory postsynaptic current (mEPSC) frequency. Conversely, when CYFIP1 is conditionally knocked out from excitatory neocortical pyramidal cells, inhibitory synaptic components are upregulated and mIPSC amplitude is significantly increased. Thus, altered gene dosage of CYFIP1 disrupts inhibitory synaptic structure, leading to altered neuronal inhibition. Our data support a role for CYFIP1 in regulating synapse number and the E/I balance and highlights a mechanism that may contribute to the neurological deficits observed in 15q11.2 CNV-associated neuropsychiatric conditions.

## Results

### CYFIP Proteins Are Enriched at Inhibitory Synapses

**Figure 1:**
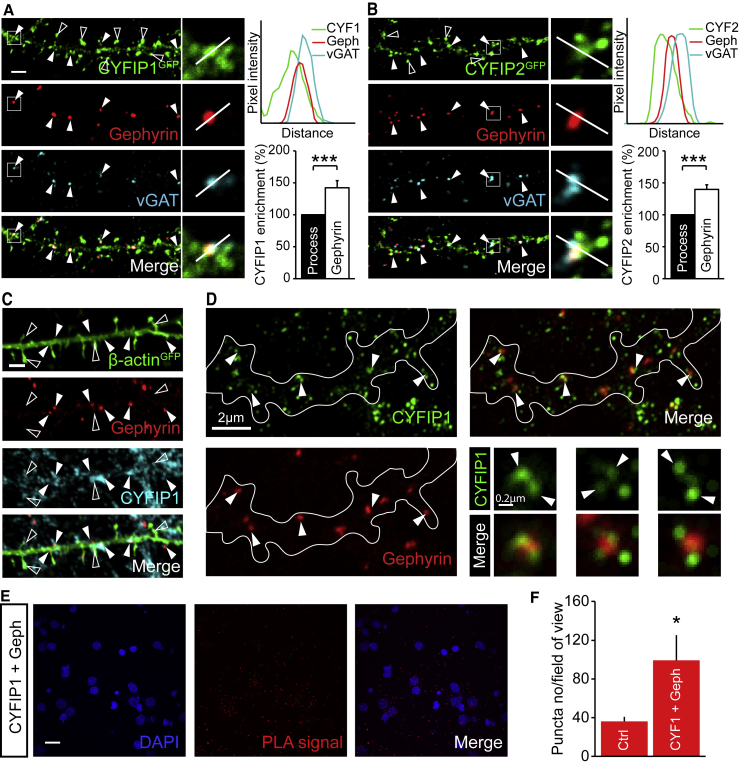
CYFIP1 and CYFIP2 Are Present at Inhibitory Synapses (A and B) Confocal images show cultured hippocampal neurons transfected with CYFIP1^GFP^ (A) or CYFIP2^GFP^ (B) and immunolabelled for VGAT and gephyrin, respectively. CYFIP1^GFP^ and CYFIP2^GFP^ clusters colocalized with the inhibitory synaptic markers (arrowheads) and are also present at dendritic spines (open arrowheads). Graphs show line scans through clusters (top) and quantification of CYFIP1^GFP^ and CYFIP2^GFP^ fluorescence intensity at gephyrin puncta compared to the total process (bottom) (CYFIP1: 42.4 ± 11.2% increase, p < 0.0001; CYFIP2: 39.8 ± 7.3% increase, p = 0.0002; n = 33–42 processes from 9 cells from 3 independent preparations; Wilcoxon signed rank test). Scale bar, 2 μm. (C) Endogenous CYFIP1 colocalizes with gephyrin (filled arrowheads) and is also present in dendritic spines (open arrowheads) in hippocampal neurons transfected with the cell fill actin^GFP^. Scale bar, 2 μm. (D) STED images of endogenous CYFIP1 and gephyrin. Arrowheads show CYFIP1 nanoclusters at gephyrin puncta. Scale bar, 2 μm; zoom scale bar, 0.2 μm. (E and F) Example images (E) and puncta quantification (F) of proximity ligation assay (PLA) on hippocampal neurons using antibodies to CYFIP1 and gephyrin compared to single CYFIP1 antibody control conditions (control: 36.1 ± 4.7; CYFIP1 and gephyrin: 99.3 ± 26.1; n = 14 cells from 3 preparations; p = 0.0217; Mann-Whitney). Scale bar, 20 μm. ^∗^p < 0.05; ^∗∗∗^p < 0.001. Bars indicate mean, and error bars indicate SEM.

While CYFIP1/2 enrichment at excitatory synapses has been previously shown (Pathania et al., 2014, De Rubeis et al., 2013), nothing is known regarding their localization to inhibitory synapses. Using immunofluorescence and confocal imaging, we examined CYFIP1 and CYFIP2 subcellular distribution in cultured neurons. CYFIP1^GFP^ and CYFIP2^GFP^ exhibited a non-uniform distribution along dendrites appearing to be selectively targeted to punctate clusters in dendritic shafts in addition to the previously reported localization of CYFIP1/2 to spine heads (Pathania et al., 2014) (Figures 1A and 1B). Labeling with inhibitory presynaptic and postsynaptic markers VGAT and gephyrin, respectively, revealed that clusters of CYFIP1^GFP^ and CYFIP2^GFP^ in dendritic shafts could be found colocalized with gephyrin opposed to VGAT-labeled inhibitory terminals (Figures 1A and 1B). This can also be seen in the line scan of the zoom images where fluorescence intensity is plotted against distance. Indeed, quantitative image analysis revealed a ∼40% enrichment of CYFIP1^GFP^ and CYFIP2^GFP^ fluorescence at gephyrin clusters compared to the total process. Endogenous CYFIP1 was also found to be highly enriched at inhibitory synapses and colocalized with gephyrin clusters (Figure 1C). To further explore the distribution of CYFIP1 within inhibitory postsynaptic sites, we carried out stimulated emission depletion (STED) microscopy to resolve this sub-synaptic compartment (Vicidomini et al., 2011). Interestingly, STED imaging performed on neurons labeled with antibodies to endogenous CYFIP1 and gephyrin revealed the presence of small CYFIP1 nanoclusters forming around gephyrin sub-synaptic domains (Figure 1D). To further investigate the intimate association of CYFIP1 with the inhibitory postsynaptic scaffold, we carried out a proximity ligation assay (PLA) (López-Doménech et al., 2018, Norkett et al., 2016). PLA detects interactions between endogenous proteins in fixed samples, giving a fluorescent readout after incubation with relevant primary antibodies, ligation, and amplification steps. The significant 2.7-fold increase in PLA puncta on neurons labeled with antibodies to endogenous CYFIP1 and gephyrin indicated an intramolecular distance of <40 nm and demonstrates that CYFIP1 can complex with gephyrin in hippocampal neurons (Figures 1E and 1F). These data indicate that CYFIP proteins can be found enriched at inhibitory synapses where they can intimately associate with the gephyrin scaffold.

### Upregulating CYFIP1 or CYFIP2 Expression Disrupts Inhibitory Synaptic Structure and Alters the Excitatory-to-Inhibitory Synaptic Ratio

**Figure 2:**
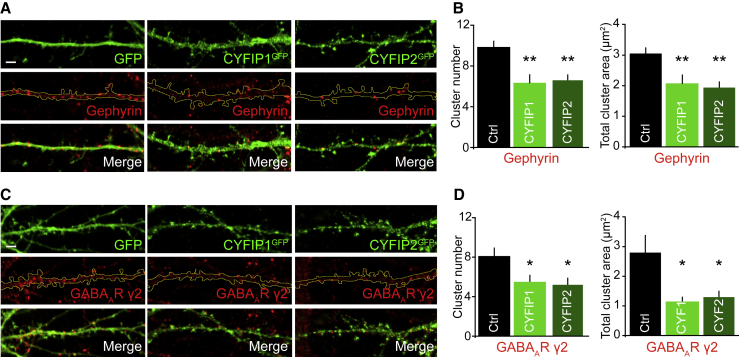
The Effect of Increased CYFIP1 and CYFIP2 Gene Dosage on Inhibitory Synaptic Structure (A) Representative confocal images of hippocampal neurons transfected with CYFIP1^GFP^, CYFIP2^GFP^, or GFP control for 4 days before labeling at DIV14 with gephyrin antibodies. Scale bar, 2 μm. (B) Gephyrin cluster analysis showing a significant decrease in gephyrin cluster number and area upon CYFIP1^GFP^ or CYFIP2^GFP^ overexpression (cluster number: from 9.9 ± 0.6 to 6.3 ± 0.9 for CYFIP1 and 6.6 ± 0.6 for CYFIP2; cluster area: from 3.1 ± 0.2 μm^2^ to 2.1 ± 0.3 μm^2^ for CYFIP1 and 1.9 ± 0.2 μm^2^ for CYFIP2; n = 20 cells from 4 preparations; Kruskal-Wallis one-way ANOVA, Dunn’s post hoc multiple comparisons). (C) Representative confocal images of hippocampal neurons transfected as in (A) and surface labeled with GABA_A_R-γ2 subunit antibodies. Scale bar, 2 μm. (D) Cluster analysis of GABA_A_R-γ2 surface puncta showing a decrease in cluster number and area upon CYFIP1^GFP^ or CYFIP2^GFP^ overexpression (cluster number: from 8.1 ± 0.9 to 5.4 ± 0.8 for CYFIP1 and 5.2 ± 0.7 for CYFIP2; cluster area: from 2.8 ± 0.6 μm^2^ to 1.1 ± 0.2 μm^2^ for CYFIP1 and 1.2 ± 0.2 μm^2^ for CYFIP2; n = 25 cells from 4 preparations; Kruskal-Wallis one-way ANOVA, Dunn’s post hoc multiple comparisons). ^∗^p < 0.05; ^∗∗^p < 0.01. Bars indicate mean, and error bars indicate SEM.

Increased CYFIP1 copy number is linked to neurodevelopmental alterations including ASD, but the impact of increased CYFIP1 or CYFIP2 expression on synaptic function remains poorly understood. Given CYFIP1/2 enrichment at inhibitory synapses, we investigated the impact of their upregulation on inhibitory synapse number and area. Cultured neurons were transfected for 4 days with CYFIP1^GFP^ or CYFIP2^GFP^ before being fixed at day *in vitro* 14 (DIV14) and labeled with an antibody against gephyrin as a marker for inhibitory synapses. Quantification revealed that gephyrin cluster number and total immunolabeled area was significantly reduced in both CYFIP1- and CYFIP2-overexpressing cells (Figures 2A and 2B). Consistent with this, the total number and area of surface GABA_A_R clusters, labeled with an antibody raised to an extracellular epitope in the synaptically enriched GABA_A_R-γ2 subunit, were also significantly reduced (Figures 2C and 2D).

**Figure 3:**
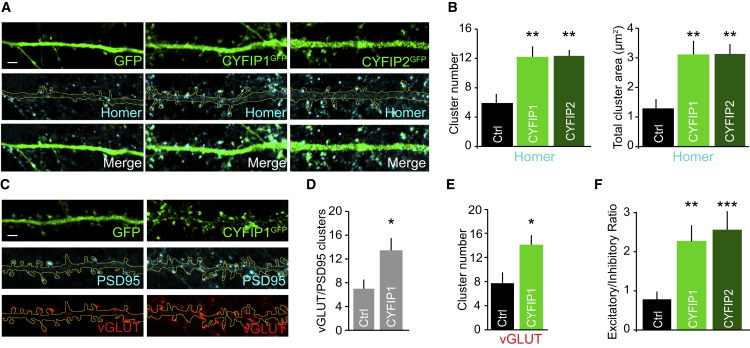
Increased Expression of CYFIP1 and CYFIP2 Alters the Ratio of Excitatory to Inhibitory Synapses (A) Confocal images of hippocampal neurons transfected with CYFIP1^GFP^, CYFIP2^GFP^, or GFP control for 4 days before labeling at DIV14 with an antibody to the excitatory postsynaptic protein homer. Scale bar, 2 μm. (B) Cluster analysis shows a significant increase in homer cluster number and area upon CYFIP1^GFP^ or CYFIP2^GFP^ overexpression (cluster number: from 5.9 ± 1.2 to 12.1 ± 1.5 for CYFIP1 and 12.3 ± 1.1 for CYFIP2; cluster area: from 1.3 ± 0.3 μm^2^ to 3.1 ± 0.5 μm^2^ for CYFIP1 and 3.1 ± 0.3 μm^2^ for CYFIP2; n = 17 cells from 3 preparations; Kruskal-Wallis one-way ANOVA, Dunn’s post hoc multiple comparisons). (C) CYFIP1^GFP^-overexpressing hippocampal neurons labeled with antibodies to VGLUT and PSD95. Scale bar, 2 μm. (D and E) Cluster analysis revealed a significant increase in total number of excitatory synapses identified as VGLUT/PSD95-positive puncta (D) and VGLUT cluster number (E) upon CYFIP1^GFP^ overexpression (total synapses: from 7 ± 1.6 to 13.6 ± 2; VGLUT number: from 7.7 ± 1.8 to 14 ± 1.7; n = 15–16 cells from 3 preparations; p = 0.018 and 0.016; Student’s t test). (F) The excitatory/inhibitory synaptic ratio quantified from transfected labeled with antibodies to homer and GABA_A_R-γ2 as markers for excitatory and inhibitory synapses respectively (E/I ratio: from 0.8 ± 0.2 to 2.3 ± 0.4 for CYFIP1 and 2.6 ± 0.5 for CYFIP2; n = 17 cells from 3 preparations; Kruskal-Wallis one-way ANOVA, Dunn’s post hoc multiple comparisons). ^∗^p < 0.05, ^∗∗^p < 0.01, ^∗∗∗^p < 0.001. Bars indicate mean, and error bars indicate SEM. See also Figure S1.

Remarkably, when neurons were labeled with an antibody to the excitatory postsynaptic density (PSD) scaffold protein homer to label excitatory synapses, the opposite effect was observed. Notably, the total number and area of homer clusters along dendrites were significantly increased in CYFIP1/2-overexpressing cells (Figures 3A and 3B). To determine whether the CYFIP1 overexpression-dependent increase in excitatory postsynapse number correlated with an increase in functional synapses, we analyzed the number of innervated excitatory synapses along the dendritic region, considered as the number of overlapping VGLUT-labeled presynaptic and PSD95-labeled postsynaptic puncta. Innervated synapses were significantly increased in cells overexpressing CYFIP1 compared to control, and consistent with this, the number of presynaptic VGLUT clusters were also enhanced (Figures 3C–3E). Significantly more excitatory synapses were found on both the dendritic shaft and spines in CYFIP1-overexpressing cells compared to control, which resulted in an increased proportion of the total number of synapses present on the shaft and a decreased ratio of synapses on the spine versus the shaft (Figures S1A–S1D). Interestingly, there was no change in spine density along dendrites, although spine morphology was altered with significantly more long thin and mushroom spines in cells overexpressing CYFIP proteins (Figures S1E–S1G).

Finally, we examined the ratio of inhibitory and excitatory synaptic clusters along dendrites in CYFIP1- or CYFIP2-overexpressing cells compared to control using antibodies against the GABA_A_R-γ2 subunit and homer, respectively. We observed a striking shift in the balance of excitatory and inhibitory synaptic puncta along dendrites upon CYFIP1/2 overexpression, which led to a significant increase in the E/I ratio (Figure 3F). Taken together, these results reveal that CYFIP protein overexpression differentially alters excitatory and inhibitory synapse number, disrupting the E/I synapse ratio.

### Disrupted Inhibitory and Excitatory Synaptic Activity in Neurons Overexpressing CYFIP1

**Figure 4:**
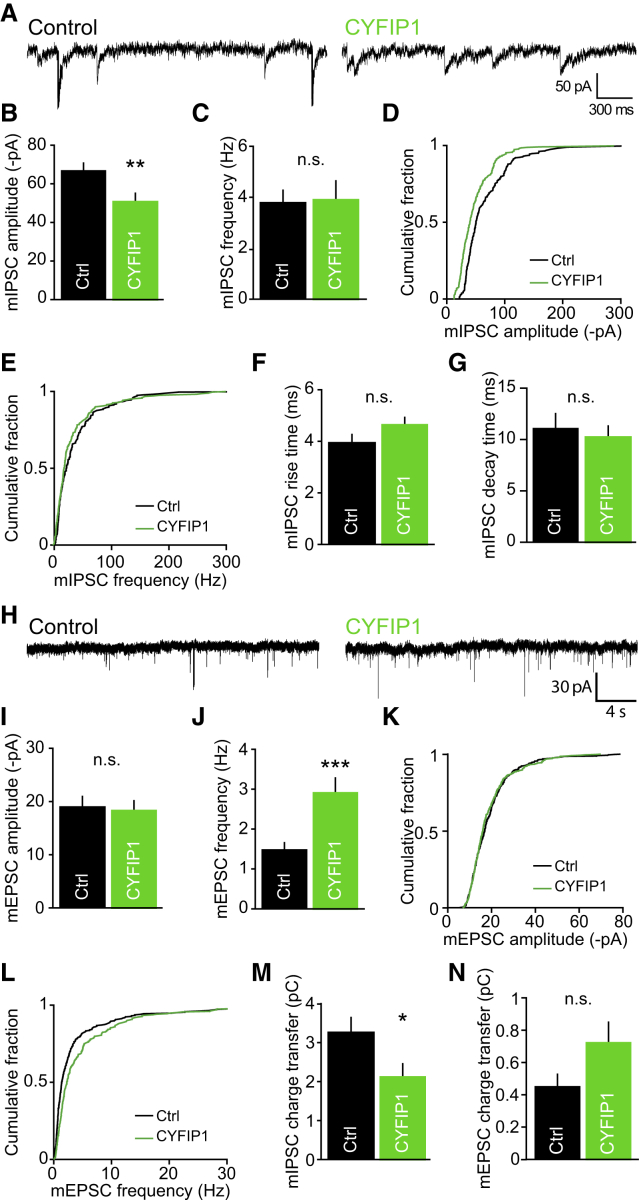
Increased CYFIP1 Gene Dosage Disrupts Inhibitory and Excitatory Synaptic Transmission (A) Representative traces of miniature inhibitory postsynaptic currents (mIPSCs) recorded from control GFP (Ctrl) and CYFIP1-overexpressing cultured hippocampal neurons at DIV14–DIV16. (B and C) Pooled data of mIPSCs showing neurons transfected with CYFIP1 have a reduction in (B) mean mIPSC amplitude but no change in (C) mean mIPSC frequency (mIPSC amplitude: from 66.9 ± 3.8 to 50.7 ± 3.6 -pA, p = 0.0062; frequency: from 3.8 ± 0.5 to 3.9 ± 0.7 Hz; p = 0.92, n.s.; all n = 10 cells from 3 preparations; Student’s t test). (D and E) Cumulative frequency graphs of mIPSC (D) amplitude and (E) frequency. (F) Graph of mIPSC rise time kinetics (from 4 ± 0.3 to 4.6 ± 0.3 ms; n = 9 cells from 3 preparations; p = 0.0623, n.s.; Mann-Whitney). (G) Graph of mIPSC decay time kinetics (from 11.1 ± 1.4 to 10.2 ± 1.1 ms; n = 10–11 cells from 3 preparations; p = 0.618, n.s.; Student’s t test). (H) Representative traces of miniature excitatory postsynaptic currents (mEPSCs) recorded from CYFIP1 or GFP control (Ctrl) transfected neurons. (I and J) Pooled data of mEPSCs showing neurons transfected with CYFIP1 have no difference in (I) mean mEPSC amplitude but a significant increase in (J) mean mEPSC frequency compared with control (mEPSC amplitude: from 19.0 ± 1.7 to 17.0 ± 1.4 -pA; p = 0.367, n.s.; frequency: from 1.5 ± 0.1 to 2.9 ± 0.4 Hz, p = 0.0003; n = 14–20 cells from 3 preparations; Student’s t test). (K and L) Cumulative frequency graphs of mEPSC (K) amplitude and (L) frequency. (M) Quantification of mIPSC total charge transfer (from 3.3 ± 0.4 to 2.1 ± 0.3 pC; n = 9 cells from 3 preparations; p = 0.0341; Student’s t test). (N) Quantification of mEPSC total charge transfer (mEPSC charge transfer: from 0.45 ± 0.1 to 0.7 ± 0.1 pC; n = 10–11 cells from 3 preparations; p = 0.1307, n.s.; Mann-Whitney). ^∗^p < 0.05, ^∗∗^p < 0.01, ^∗∗∗^p < 0.001. Bars indicate mean, and error bars indicate SEM. See also Figure S2.

We further addressed whether increased CYFIP dosage can also directly affect inhibitory and excitatory transmission in neurons, focusing on CYFIP1 as the gene has been more robustly associated with neurodevelopmental disorders. Whole-cell recordings were performed to measure inhibitory and excitatory transmission in neurons overexpressing CYFIP1 and co-expressing GFP (Figures S2A–S2C) (Kim et al., 2011). Analysis of mIPSCs from CYFIP1-overexpressing cells revealed a significant ∼25% decrease in mIPSC amplitude but no change in frequency compared to control neurons expressing GFP alone (Figures 4A–4C). The decreased mean mIPSC amplitude can be seen in the representative traces and in the leftward shift of the cumulative probability plot (Figures 4D and 4E). Overexpression of CYFIP1 had no effect on mIPSC kinetics (Figures 4F and 4G). Conversely, when we analyzed mEPSCs, we observed no change in mEPSC amplitude but saw a robust and significant increase in mEPSC frequency (Figures 4H–4J). Again, this finding can be observed in both the example traces and the shift toward the right in the cumulative probability plot of mEPSC frequency (Figures 4K and 4L). The kinetics of mEPSCs was unchanged (Figures S2D and S2E). Finally, the mean total charge transfer (a parameter that reflects both the amplitude and frequency of miniature synaptic events) for mIPSCs was significantly decreased in CYFIP1-overexpressing cells, while mEPSC charge transfer showed a trend toward an increase (Figures 4M and 4N). These data demonstrate that CYFIP1 overexpression not only alters synapse numbers but also results in functional deficits in synaptic transmission, resulting in an imbalance of excitation and inhibition.

### Decreased CYFIP1 Gene Dosage Alters Neuronal and Dendritic Spine Morphology

**Figure 5:**
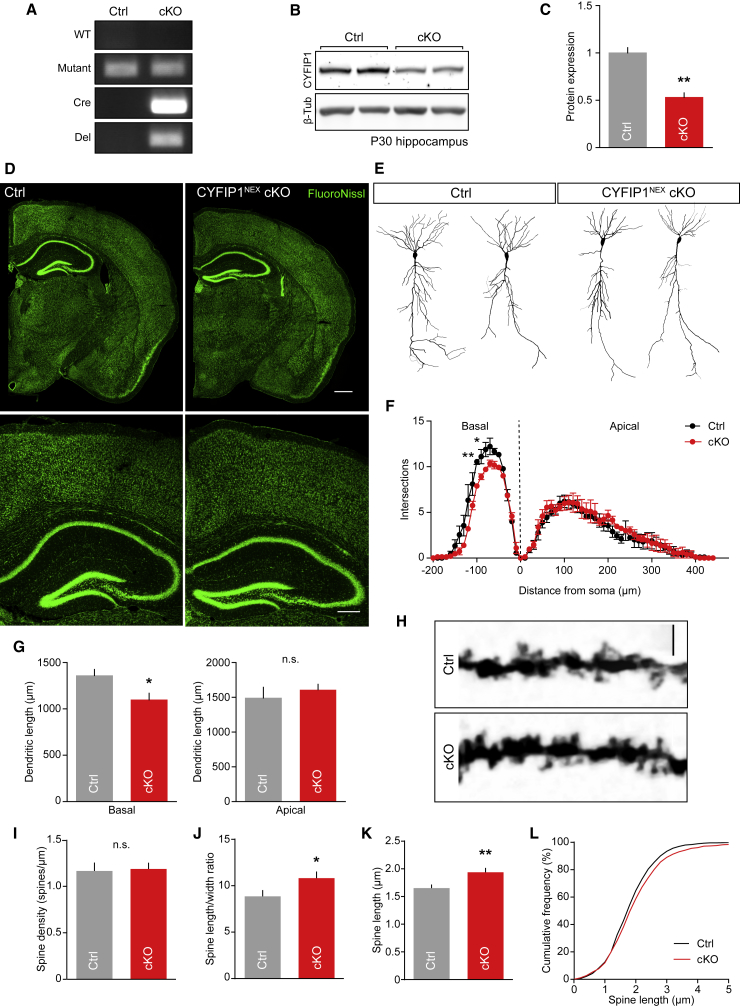
Loss of CYFIP1 Expression in Principal Cells of the Neocortex Alters Hippocampal Cell Morphology (A) PCR genotyping of CYFIP1^NEX^ conditional knockout (cKO) animals generated from the KO-first strategy. CYFIP1 floxed animals were crossed with mice expressing cre recombinase under the Nex promoter. Animals were genotyped with wild-type (WT), mutant, cre recombinase (Cre), and deletion (Del) primers. See also Figure S3. (B and C) Western blot (B) and quantification (C) of floxed control (Ctrl) and CYFIP1^NEX^ conditional KO (cKO) P30 hippocampal brain lysates (from 1 ± 0.1 to 0.5 ± 0.05; n = 3 animals per condition; p = 0.0033, Student’s t test). (D) Fluoro-Nissl staining of control floxed (Ctrl) and CYFIP1^NEX^ cKO P30 mouse coronal brain sections. Scale bar, 500 μm; zoom, 250 μm. (E) Example reconstructions of CYFIP1^NEX^ cKO and floxed littermate control (Ctrl) Golgi-stained P30 CA1 neurons. (F) Sholl analysis of Golgi-stained neurons to measure dendritic complexity (Basal dendrites, −100 μm: p < 0.05; −120 μm: p < 0.01; two-way ANOVA, Bonferroni’s multiple comparisons). (G) Total dendritic length of Golgi-stained neurons (dendritic length: basal, from 1,360 ± 65.9 to 1,099 ± 70.5 μm; p = 0.0178; apical, from 1,490 ± 153.5 μm to 1,609 ± 78 μm; p = 0.46, n.s.; n = 9–13 reconstructed cells from 3 animals per genotype; Student’s t test). (H) Example dendrites and dendritic spines of CYFIP1^NEX^ cKO (cKO) and floxed littermate control (Ctrl) Golgi-stained P30 CA1 neurons. Scale bar, 5 μm. (I–K) Dendritic spine analysis revealed no change in spine density (I) between genotypes but an increase in spine length/width ratio (J) in CYFIP1^NEX^ cKO neurons as a result of an increase in dendritic spine length (K) (spine density: from 1.2 ± 0.1 to 1.2 ± 0.1 spines/μm; p = 0.32, n.s.; length/width ratio: from 8.9 ± 0.7 to 10.8 ± 0.7, p = 0.0407; length: from 1.7 ± 0.1 μm to 1.9 ± 0.1 μm, p = 0.02; n = 45 dendritic processes from 3 animals per genotype, Student’s t test). (L) Cumulative frequency graph of dendritic spine length. ^∗^p < 0.05; ^∗∗^p < 0.01. Bars indicate mean, and error bars indicate SEM.

*CYFIP1* can also undergo microdeletion, but due to the embryonic lethality of constitutive CYFIP1 knockout (KO), the impact of deleting all CYFIP1 in the brain remains undetermined (Pathania et al., 2014). To circumvent this and study cell-type-specific effects of CYFIP1 deletion, we generated a conditional KO (cKO) mouse line selectively lacking CYFIP1 in forebrain excitatory neurons using a Nex-Cre driver line (Goebbels et al., 2006, Skarnes et al., 2011). This allowed us to determine the impact of deleting CYFIP1 from embryonic day 12 onward on neuronal development specifically in excitatory cells of the forebrain (Figures 5A and S3A). CYFIP1^NEX^ cKO animals were viable until adulthood with no obvious abnormalities. Western blotting of post-natal day 30 (P30) hippocampal brain lysates with a CYFIP1-specific antibody revealed a robust reduction of CYFIP1 expression in CYFIP1^NEX^ cKO mice compared to control floxed animals (Figures 5B and 5C). Remaining CYFIP1 expression detected in western blots presumably comes from CYFIP1 in other cell populations such as interneurons and glia. Fluoro-Nissl labeling of thin brain sections revealed that CYFIP1^NEX^ cKO mice did not show any gross morphological abnormalities in neocortical and hippocampal brain structure when compared to control (Figure 5D).

CYFIP1 haploinsufficiency in constitutive CYFIP1 heterozygous KO mice led to decreased dendritic complexity and altered dendritic spine maturation both *in vitro* and *in vivo* (Pathania et al., 2014). Therefore, we initially assessed dendritic morphology in hippocampal neurons from CYFIP1^NEX^ cKO mice. Golgi-stained CA1 neurons analyzed from P30 CYFIP1^NEX^ cKO brains showed significantly less dendritic complexity in the basal compartment compared to neurons analyzed from littermate control tissue. Consistent with this, total basal dendritic length was reduced; however, branch point number was unchanged (Figures 5E–5G). While spine density was unchanged in CYFIP1^NEX^ cKO neurons, there was a significant increase in the spine length and length-to-width ratio consistent with the spine phenotypes previously reported upon constitutive CYFIP1 heterozygous KO (Pathania et al., 2014) (Figures 5H–5L). Thus, these data illustrate that the CYFIP1^NEX^ cKO mice show similar deficits in dendrite morphology and spine maturation to those described for a CYFIP1 haploinsufficient model and support these effects to be cell autonomous to the CA1 pyramidal cells.

### Postsynaptic Loss of CYFIP1 Increases Inhibitory Synapse Size and Strength

**Figure 6:**
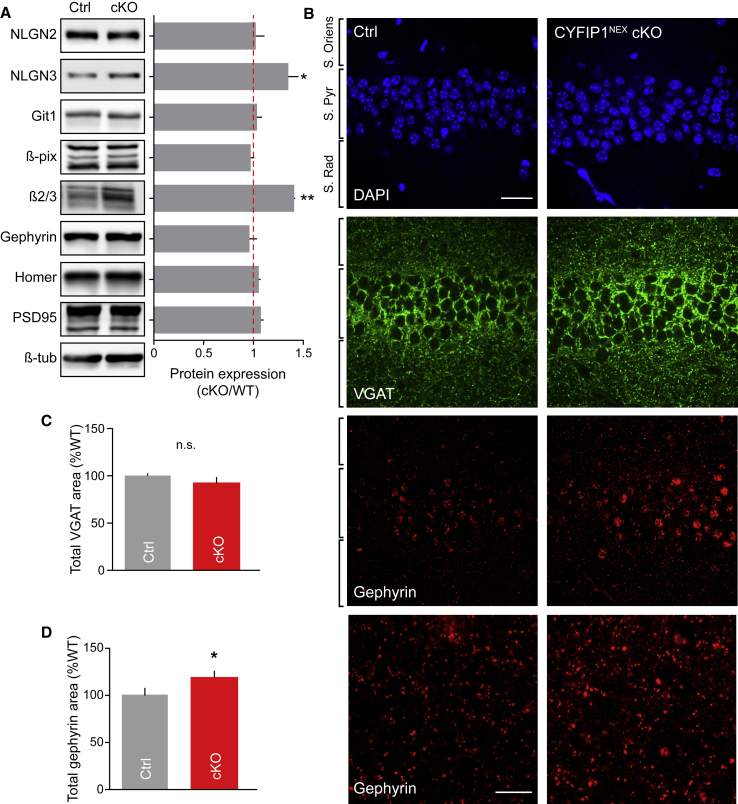
Decreased CYFIP1 Gene Dosage Alters Expression of Inhibitory Scaffold Molecules and Inhibitory Synaptic Structure *In Vivo* (A) Western blot analysis displaying protein expression ratios of synaptic proteins from control (Ctrl) and CYFIP1^NEX^ conditional KO (cKO) P30 hippocampal brain lysates (neuroligin 2 [NLGN2]: 1.02 ± 0.09; neuroligin 3 [NLGN3]: 1.35 ± 0.1, p = 0.0286; Git1: 1.03 ± 0.05; β-pix: 0.97 ± 0.04; GABA_A_R β2/3: 1.41 ± 0.01, p = 0.0017; gephyrin: 0.96 ± 0.07; homer: 1.05 ± 0.02; PSD95: 1.07 ± 0.03; n = 3 animals per condition; Student’s t test). (B) Confocal images of adult control (Ctrl) and CYFIP1^NEX^ cKO hippocampal brain sections immunolabelled with antibodies to VGAT and gephyrin, co-stained with DAPI. Scale bar, 25 μm; zoom, 10 μm. (C and D) Normalized total cluster area quantification of CYFIP1^NEX^ cKO (cKO) tissue as a percentage of floxed control (Ctrl) showing no change in (C) VGAT cluster area and an increase in (D) gephyrin cluster area (VGAT: from 100 ± 2.4% to 94 ± 5.2%; p = 0.336, n.s.; gephyrin: from 100 ± 6.9% to 119 ± 6.1%; p = 0.0473; n = 18 hippocampal regions from 3 animals per genotype; Student’s t test) in CYFIP1 cKO tissue compared to control. ^∗^p < 0.05; ^∗∗^p < 0.01. Bars indicate mean, and error bars indicate SEM.

To further explore the impact of CYFIP1 deletion on synaptic components, we probed hippocampal lysates from P30 control and CYFIP1^NEX^ cKO brains with antibodies to key molecular components of the inhibitory and excitatory PSDs. Interestingly, while the levels of key excitatory postsynaptic proteins including homer and PSD95 were unchanged, we observed a significant increase in the levels of the inhibitory GABA_A_R-β2/3 subunits and the ASD-associated neuroligin 3 adhesion molecule, which can be found at both inhibitory and excitatory postsynapses (Figure 6A). CYFIP1 loss of function may therefore have an opposite effect to that of upregulation, causing an increase in inhibitory synapse stability. To validate this, we carried out immunohistochemistry on thin hippocampal sections taken from P30 control floxed and CYFIP1^NEX^ cKO brains. Sections were labeled with antibodies to VGAT and gephyrin to report inhibitory presynaptic and postsynaptic compartments and DAPI to indicate cell bodies. Quantification in the *stratum pyramidale* layer of the hippocampus revealed a significant increase in normalized gephyrin cluster area in cKO tissue compared to control while VGAT cluster area was unchanged (Figures 6B–6D). These data highlight that loss of CYFIP1 *in vivo* in glutamatergic principal cells results in an increase in inhibitory synapse size and the levels of inhibitory synaptic proteins.

**Figure 7:**
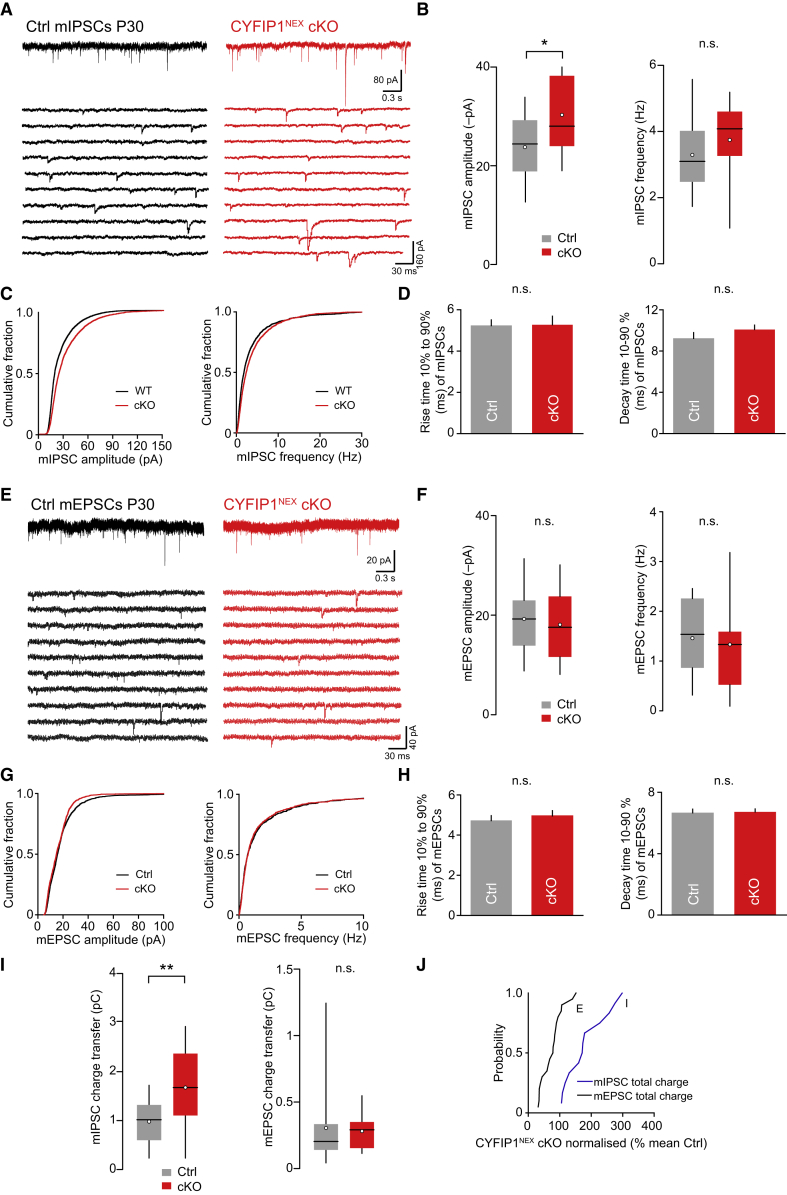
Postsynaptic Loss of CYFIP1 *In Vivo* Increases Inhibitory Synaptic Function (A) Representative recordings of miniature inhibitory postsynaptic currents (mIPSCs) (–70 mV) in CA1 pyramidal cells from P28–P34 control floxed (left) and CYFIP1^NEX^ cKO mice (right). Lower panels are representative sections of recordings (contiguous 0.3-s segments). (B) Pooled data showing increase mIPSC mean amplitude in CYFIP1^NEX^ cKO mice (cKO) (from 23.8 ± 1.8 to 30.3 ± 2.2 -pA; n = 13–14 cells; p = 0.0288) but no change in frequency (from 3.3 ± 0.3 to 3.7 ± 0.3 Hz; n = 13–15 cells; p = 0.324, n.s.) All Student’s t test. Box-and-whisker plots indicate median (line), 25th to 75th percentiles (box), and range of data within 1.5× interquartile range (IQR) of box (whiskers) and mean (open circles). (C) Cumulative frequency graphs of mIPSC amplitude (left) and frequency (right). (D) Graphs of mIPSC kinetics showing no change in rise and decay time (rise time: from 5.2 ± 0.3 to 5.3 ± 0.4 ms; n = 13–14 cells; p = 0.935, n.s.; decay time: 9.2 ± 0.6 to 10.1 ± 0.4 ms; n = 13–15 cells; p = 0.247, n.s.; both Student’s t test). (E) Representative recordings of miniature excitatory postsynaptic currents (mEPSCs) (–70 mV) in CA1 pyramidal cells from P28–P34 control floxed (left) and CYFIP1^NEX^ cKO mice (right). Lower panels are representative sections of recordings (contiguous 0.3-s segments). (F) Pooled data showing no change in mEPSC mean amplitude (from 19.2 ± 1.4 to 18 ± 1.4 -pA; n = 21–22 cells; p = 0.549, n.s.) and frequency between control (Ctrl) and CYFIP1 cKO mice (cKO) (from 1.5 ± 0.2 to 1.3 ± 0.2 Hz; n = 18–20 cells; p = 0.565, n.s.). All Student’s t test. Box-and-whisker plots indicate median (line), 25th to 75th percentiles (box), and range of data within 1.5× IQR of box (whiskers) and mean (open circles). (G) Cumulative frequency graphs of mEPSC amplitude (left) and frequency (right). (H) Graphs of mEPSC kinetics showing no change in rise and decay time (rise time: from 4.7 ± 0.2 to 5 ± 0.3 ms; n = 22 cells; p = 0.527, n.s.; decay time: from 6.7 ± 0.2 to 6.7 ± 0.2 ms; n = 22 cells; p = 0.8415, n.s.; both Student’s t test). (I) Pooled data showing an increase in mIPSC mean charge transfer in CYFIP1^NEX^ cKO (cKO) mice compared to floxed control (Ctrl) (from 1 ± 0.2 to 1.7 ± 0.2 pC; n = 13–14 cells; p = 0.0067) but no change in mEPSC mean charge transfer (from 0.3 ± 0.1 to 0.3 ± 0.03 pC; n = 18–20 cells; p = 0.753, n.s.) All Student’s t test. Box-and-whisker plots indicate median (line), 25th to 75th percentiles (box), and range of data within 1.5× IQR of box (whiskers) and mean (open circles). (J) The probability curve of mean mEPSC and mIPSC charge transfer in CYFIP1^NEX^ cKO mice as a percentage of control mice highlighting the imbalance between excitation and inhibition in CYFIP1^NEX^ cKO animals. ^∗^p < 0.05; ^∗∗^p < 0.01. Bar graph bars indicate mean, and error bars indicate SEM. See also Figure S4.

Finally, we investigated whether the changes in inhibitory synapses observed in CYFIP1^NEX^ cKO mice translated into a functional effect on synaptic transmission. We examined mIPSCs in acute hippocampal slices from control and CYFIP1^NEX^ cKO P28–P34 mice, in which CA1 pyramidal cells could be identified unambiguously. Recordings from these cells showed that deletion of CYFIP1 resulted in a significant increase in mIPSC amplitude consistent with a shift to the right in the cumulative frequency plot of mIPSC amplitude (Figures 7A–7C). This was not observed with acute short hairpin RNA (shRNA) knockdown of CYFIP1 from cultured neurons (Figures S4A–S4D). No change was observed in mIPSC frequency and mIPSC rise and decay time between control and CYFIP1^NEX^ cKO CA1 pyramidal neurons (Figures 7A–7D). Importantly, CYFIP1 deletion had no effect on AMPA receptor (AMPAR)-mediated mEPSCs (Figures 7E–7H), confirming a selective effect on synaptic inhibition in P28–P34 animals. Lastly, we measured the total charge transfer mediated by both inhibitory and excitatory postsynaptic currents. This showed that mIPSC charge transfer was increased by ∼70% in CYFIP1-deleted cells compared to control, while mEPSC charge transfer was unchanged (Figure 7I). The probability curve of mIPSC and mEPSC charge transfer from CYFIP1^NEX^ cKO neurons normalized to control demonstrates the resultant imbalance between inhibitory and excitatory transmission observed with loss of CYFIP1 expression (Figure 7J). Altogether, this highlights the necessity of CYFIP1 for correct synaptic inhibition, as its absence during development dramatically impacts inhibitory synapse integrity and the strength of inhibition.

## Discussion

Alterations in E/I balance are implicated in neuropsychiatric disorders including ASD and SCZ (Foss-Feig et al., 2017), but how this may be caused by genetic variation is poorly understood. Here, we report that the ASD- and SCZ-associated protein CYFIP1 is localized to inhibitory synapses and regulates the balance between neuronal excitation and inhibition. CYFIP1 overexpression increases the excitatory-to-inhibitory synaptic ratio consistent with an increase in mEPSC frequency and decrease in mIPSC amplitude. In contrast, CYFIP1 loss had the opposite effect on neuronal inhibition, leading to increased inhibitory postsynaptic clustering, enhanced expression of neuroligin 3 and GABA_A_R β-subunits, and an increase in mIPSC amplitude *in vivo*. Our data provide strong support for altered inhibition and disruption in E/I balance being a pathological consequence of CYFIP1 CNV and points toward disruption in inhibitory synaptic structure and function as being part of the underlying mechanism.

CYFIP1 was previously shown to be enriched at excitatory synapses where it can regulate F-actin dynamics (Pathania et al., 2014, De Rubeis et al., 2013), protein translation (Napoli et al., 2008), and dendritic spine structural plasticity (Pathania et al., 2014). Our data show that increased CYFIP1 dosage in hippocampal cultures increased VGLUT and homer-positive cluster number and consequently total cluster area and thus increased excitatory synapse number and mEPSC frequency. Notably, a high proportion of these synapses appeared on the dendritic shaft compared to spines. This is perhaps due to the molecular and spatial confinement present within the spines as we detect more long thin spines on CYFIP1-overexpressing cells, an immature subtype of spines that has been repeatedly identified in rodent models and patients with ASD (Phillips and Pozzo-Miller, 2015) as well as a transgenic mouse overexpressing *Cyfip1* (Oguro-Ando et al., 2015). Importantly, our transfection approach results in CYFIP1 overexpression in a sparse population of neurons, whose vast majority of presynaptic inputs will be from wild-type (WT) untransfected cells. Thus, the mEPSC frequency increase we observe likely reflects a postsynaptically driven increase in excitatory synapse number, rather than any presynaptic effect on excitability or release probability (Hsiao et al., 2016).

In addition, we demonstrate that CYFIP1 and CYFIP2 are also enriched at inhibitory synapses and that CYFIP1 interacts with the inhibitory postsynaptic scaffold gephyrin, supporting its intimate association with the inhibitory postsynaptic density. STED imaging revealed CYFIP1 and gephyrin are in closely adjacent clusters consistent with the sub-synaptic localization of other inhibitory synaptic enriched proteins (Woo et al., 2013) and thus is well-positioned to influence these proteins. In contrast to the effects on excitatory synapses, increased CYFIP1 dosage reduced inhibitory postsynaptic cluster area and decreased amplitude of inhibitory transmission likely due to a loss of surface γ2-subunit-containing synaptic GABA_A_R clusters. Taken together, upregulation of CYFIP1 expression impacts excitatory and inhibitory synapses and would likely lead to altered E/I balance.

Interestingly, CYFIP2 overexpression phenocopied both inhibitory and excitatory alterations in synapse number and size observed with increased CYFIP1 dosage. Although CYFIP2 CNVs have yet to be reported, altered CYFIP2 function has been associated with neurological and neuropsychiatric disorders through genetic associations and expression changes (Föcking et al., 2015, Han et al., 2015, Nakashima et al., 2018). Our data suggest that increased CYFIP2 dosage could lead to altered synaptic inhibition, which may contribute to the pathology underlying CYFIP2-associated neurological disorders.

In addition to studying CYFIP1 upregulation, we also determined the impact of CYFIP1 loss on neuronal development and connectivity. Constitutive KO of CYFIP1 leads to early embryonic lethality, thus far limiting CYFIP1 loss-of-function studies to exploring the impact of CYFIP1 haploinsufficiency (Bozdagi et al., 2012, Hsiao et al., 2016, Pathania et al., 2014, De Rubeis et al., 2013). While these have been informative, it is clearly also important to establish the impact of complete loss of CYFIP1 in CNS neurons and the cell autonomy of CYFIP1 dysfunction in glutamatergic neurons, where most studies have focused. To this end, we developed a CYFIP1^NEX^ cKO where CYFIP1 was deleted from principal cells of the hippocampus and neocortex. Surprisingly, CYFIP1^NEX^ cKO resulted in relatively mild defects in dendritic branching and spine maturation in P30 CA1 pyramidal cells, similar to those previously reported upon CYFIP1 haploinsufficiency (Pathania et al., 2014), perhaps due to compensation from CYFIP2 (Han et al., 2015, Pathania et al., 2014). CYFIP1^NEX^ cKO animals did not exhibit alterations in excitatory synaptic transmission at this age consistent with reports of adult CYFIP1 haploinsufficient mice where basal excitatory synaptic transmission was unaltered (Bozdagi et al., 2012). Importantly, the cell selectivity of our model supports dendritic branching and spine alterations upon disrupted CYFIP1 expression (Oguro-Ando et al., 2015, Pathania et al., 2014, De Rubeis et al., 2013) to be primarily cell-autonomous effects.

Several lines of evidence reported here support enhanced inhibitory postsynaptic function in CYFIP1^NEX^ cKO animals. We found increased expression levels of GABA_A_R-β2/3 subunits and neuroligin 3 in the hippocampus. Furthermore, we observed an increase in gephyrin clustering and mIPSC amplitude in hippocampal CA1 neurons. Given that neurons providing inhibition to CA1 pyramidal cells are not targeted by the Nex Cre-driven CYFIP1 deletion, these effects reflect postsynaptic changes in CA1 cell function. Furthermore, the unaltered excitatory transmission in CYFIP1^NEX^ cKO mice argues against an increased excitatory drive of inhibitory interneurons. Intriguingly, acute downregulation of CYFIP1 in culture did not recapitulate the increased inhibitory synaptic size and strength we observed in CYFIP1^NEX^ cKO animals, suggesting long-term postsynaptic CYFIP1 loss is required for the observed effects.

As the majority of synaptic GABA_A_Rs require the incorporation of β2/3 subunits to form and be efficiently trafficked to the plasma membrane, higher levels of these subunits likely reflect increased numbers of synaptic GABA_A_Rs. These GABA_A_Rs are stabilized at the postsynapse by adhesion molecules including neuroligins, a family of synaptic adhesion molecules associated with neurodevelopmental disorders (Bemben et al., 2015a, Jamain et al., 2003). While neuroligins 1 and 2 are localized to excitatory and inhibitory synapses, respectively (Song et al., 1999, Varoqueaux et al., 2004), neuroligin 3 can be found at both types of synapse and interacts with gephyrin and neuroligin 2 at inhibitory synapses (Budreck and Scheiffele, 2007). By stabilizing GABA_A_Rs at synapses, upregulated neuroligin 3 may help drive the increased levels of GABA_A_R-β2/3 subunits observed and contribute to the increase in synaptic inhibition. Given its dual synaptic localization, it is intriguing that neuroligin 3 upregulation appears to only enhance synaptic inhibition in our model. However, recent work has shown that overexpression of neuroligin 3 leads to a selective increase in the strength of inhibition over excitation (Chanda et al., 2017), and ASD-associated point mutations in NLGN3 have been show to impact the E/I synaptic ratio in neurons (Tabuchi et al., 2007, Zhang et al., 2017). Taken together, these data point toward CYFIP1 having a role in synaptic development similarly to other ASD-associated synaptic and actin regulatory molecules such as Shank3, neuroligins, and drebrin (Bemben et al., 2015b, Grabrucker et al., 2011, Shirao et al., 2017).

How CYFIP1 expression levels bi-directionally regulate postsynaptic excitation and inhibition while being enriched at both synapses remains to be fully elucidated. CYFIP1 interacts with a number of proteins present at both inhibitory and excitatory synapses including Rac1, WAVE1, and FMRP that have numerous synapse-specific functions involved in actin remodeling and protein translation (Kobayashi et al., 1998, Napoli et al., 2008, Uezu et al., 2016). For instance, the GIT1-Rac1 pathway has been shown to regulate spine morphology and GABA_A_R stability and endocytosis at excitatory and inhibitory synapses, respectively (Smith et al., 2014, Wang et al., 2017, Zhang et al., 2005). Perturbed CYFIP1 expression could impact synapse-specific Rac1-dependant pathways via altered availability of Rac1-GTP. Alternatively, CYFIP1 itself may have synapse-specific interactions. CYFIP1 forms a binding surface with Abi that enables binding between the WRC and proteins containing a WIRS (WRC interacting receptor sequence) peptide motif (Chen et al., 2014, Chia et al., 2014). Interestingly, neuroligin 3 (but not neuroligin 2) contains a WIRS motif. Disrupted coupling of neuroligin 3 to the WRC upon CYFIP1 deletion might alter its trafficking, by disrupting its surface downmodulation or intracellular sorting (Anitei et al., 2010, Xu et al., 2016). Changes in surface stability or turnover of neuroligin 3 might work hand in hand with increased neuroligin 3 expression due to relief of FMRP-dependent translational repression upon CYFIP1 loss (Darnell et al., 2011, Napoli et al., 2008).

E/I balance shift can lead to deficits in network activity, disrupted information processing, and altered behaviors (Blundell et al., 2009, Gkogkas et al., 2013, Tora et al., 2017, Yizhar et al., 2011). An increase in the ratio of excitatory to inhibitory synapses as observed upon CYFIP1 upregulation is consistent with altered E/I balance in ASD and in mouse models of numerous neuropsychiatric disorders (Bateup et al., 2013, Chao et al., 2010, Gao and Penzes, 2015, Nelson and Valakh, 2015, Smith et al., 2017) and the increased risk of neuropsychiatric symptoms in some individuals with *CYFIP1* duplication. Intriguingly, patients with temporal lobe epilepsy and pilocarpine-treated rats show an upregulation of CYFIP1 expression consistent with the notion that increased CYFIP1 expression is associated with altered E/I balance and associated behaviors (Huang, 2016). Enhanced inhibition upon CYFIP1 deletion could contribute to the intellectual disability and cognitive changes reported in individuals with 15q11.2 microdeletions. Indeed, excess inhibition contributes to cognitive impairment in Down syndrome models where disrupted long-term potentiation, learning, and memory can be improved by pharmacologically targeting GABA_A_Rs (Rudolph and Möhler, 2014). Interestingly, neuroligin 3 overexpression in hippocampal pyramidal cells was also recently reported to selectively increase synaptic inhibition by somatostatin-expressing interneurons that innervate distal dendrites at the expense of perisomatic inputs from parvalbumin-expressing interneurons (Horn and Nicoll, 2018). Whether CYFIP1 deletion and concomitant neuroligin 3 upregulation could similarly alter the balance of inhibitory circuit control by these two types of interneuron remains to be determined.

Our results have established a link between altered CYFIP1 dosage, changes in synaptic inhibition and excitation, and altered E/I balance. This provides important insights into the role CYFIP proteins have in synaptic function and network activity and how CYFIP1 dysregulation in 15q11.2 CNV may impact CNS function to contribute to the development of neuropsychiatric and neurodevelopmental disorders. Furthermore, our work supports the idea that synaptic inhibition is a therapeutic target and that drugs acting on GABA_A_Rs may prove beneficial for individuals harboring CYFIP1 CNVs.

## STAR★Methods

### Key Resources Table

**Table:** 

REAGENT or RESOURCE	SOURCE	IDENTIFIER
**Antibodies**		

Rabbit anti-CYFIP1	Millipore	Cat# 07-531; RRID:AB_390148
Mouse anti-GABA_A_R-β2/3	Neuromab	Cat# 75-363; RRID:AB_2315838
Guinea-pig anti-GABA_A_R-γ2	Synaptic Systems	Cat# 224 004; RRID:AB_10594245
Mouse anti-gephyrin	Synaptic Systems	Cat# 147 011; RRID:AB_887717
Rat anti-GFP	Nacalai-Tesque	Cat# GF090R; RRID:AB_10013361
Mouse anti-GFP (N86/8)	Neuromab	Cat# 73-131; RRID:AB_10671444
Mouse anti-GIT1 (N39/B8)	Neuromab	Cat# 75-094; RRID:AB_2109991
Rabbit anti-Homer	Synaptic Systems	Cat# 160 002; RRID:AB_2120990
Rabbit anti-neuroligin2	Synaptic Systems	Cat# 129 202; RRID:AB_993011
Mouse anti-neuroligin3 (N110/29)	Neuromab	Cat# 75-158; RRID:AB_2151818
Mouse anti-PSD95 (K28/43)	Neuromab	Cat# 75-028; RRID:AB_2292909
Rabbit anti-βPIX	Millipore	Cat# 07-1450; RRID:AB_1586904
Mouse anti-βtubulin	Sigma-Aldrich	Cat# T5293; RRID:AB_477580
Rabbit anti-VGAT	Synaptic Systems	Cat# 131 003; RRID:AB_887869
Guinea-pig anti-VGLUT	Synaptic Systems	Cat# 135 304; RRID:AB_887878
Mouse anti-V5	Invitrogen	Cat# R960-25; RRID:AB_2556564
Rabbit anti-RFP	Abcam	Cat# ab62341; RRID:AB_945213
Goat anti-rabbit IgG (H+L), HRP	Jackson ImmunoResearch	Cat# 111-035-003; RRID:AB_2313567
Goat anti-mouse IgG (H+L), HRP	Jackson ImmunoResearch	Cat# 115-035-003; RRID:AB_10015289
Donkey anti-rat Alexa Fluor 488	ThermoFisher Scientific	Cat# A-21208; RRID:AB_141709
Donkey anti-rabbit Alexa Fluor 488	ThermoFisher Scientific	Cat# A-21206; RRID:AB_2535792
Donkey anti-mouse Alexa Fluor 568	ThermoFisher Scientific	Cat# A-10037; RRID:AB_2534013
Goat anti-guinea pig Alexa Fluor 568	ThermoFisher Scientific	Cat# A-11075; RRID:AB_2534119
Donkey anti-rabbit Alexa Fluor 647	ThermoFisher Scientific	Cat# A-31573; RRID:AB_2536183
Donkey anti-mouse Alexa Fluor 647	ThermoFisher Scientific	Cat# A-31571; RRID:AB_162542

**Bacterial and Virus Strains**		

One Shot TOP10 Chemically Competent E.coli	Invitrogen	Cat# C404010

**Chemicals, Peptides, and Recombinant Proteins**		

NBQX	Abcam	Cat# ab120046
AP5	Abcam	Cat# ab120003
Tetrodotoxin citrate (TTX)	Tocris	Cat# 1078
Bicuculline	Abcam	Cat# ab120108
Lipofectamine-2000	ThermoFisher Scientific	Cat # 11668027
Tissue-Tek^∗^ O.C.T. Compound	Sakura Finetek	Cat #4583
NeuroTrace™ 500/525 Green Fluorescent Nissl Stain	ThermoFisher Scientific	Cat# N21480
ProLong™ Gold Antifade Mountant	ThermoFisher Scientific	Cat# P36930
Hank’s Buffered Salt Solution (HBSS)	ThermoFisher Scientific	Cat # 14180046
1M HEPES buffer	ThermoFisher Scientific	Cat # 15630-080
2.5% Trypsin	ThermoFisher Scientific	Cat # 15090046
Minimal Essential Medium (MEM)	ThermoFisher Scientific	Cat # 31095029
Heat inactivated Horse Serum (HRS)	ThermoFisher Scientific	Cat # 26050088
Sodium pyruvate	ThermoFisher Scientific	Cat # 11360070
Glucose	ThermoFisher Scientific	Cat # A2494001
Poly-L-lysine (PLL)	Sigma-Aldrich	Cat # P6282-5MG
Neurobasal	ThermoFisher Scientific	Cat # 21103049
B27	ThermoFisher Scientific	Cat # 17504044
glutaMAX	ThermoFisher Scientific	Cat # 35050061
DMEM, high glucose	ThermoFisher Scientific	Cat # 41965039
Fetal Bovine Serum	ThermoFisher Scientific	Cat # 10082147
Penicillin/Streptomycin	ThermoFisher Scientific	Cat # 15140122

**Critical Commercial Assays**		

FD Rapid Golgi Stain Kit	FD NeuroTechnologies	Cat# PK401
Duolink® *In Situ* Red Starter Kit Mouse/Rabbit	Sigma-Aldrich	Cat# DUO92101
Gateway™ LR Clonase™ Enzyme Mix	Invitrogen	Cat# 11791019
In-Fusion® HD Cloning Plus	Takara	Cat# 638911

**Experimental Models: Cell Lines**		

COS-7	ATCC	Cat# CRL-1651

**Experimental Models: Organisms/Strains**		

Mouse: *Cyfip1*^tm2a(EUCOMM)Wtsi^	Wellcome Trust Sanger Institute	N/A
Mouse: *Cyfip1*^NEX^ cKO	This paper	N/A
Rat: Wild-type Sprague Dawley	Charles River	N/A

**Oligonucleotides**		

Genotyping primer, CAS_R1_Term (Mut^R^): TCGTGGTAT CGTTATGCGCC	(Pathania et al., 2014)	N/A
Genotyping primer, Cyfip1_234230_F (Wt^F^): TGGAAGTA ATGGAACCGAACA	(Pathania et al., 2014)	N/A
Genotyping primer, Cyfip1_234230_R (Wt^R^): GTAACTAC CTATAATGCAGACCTGAAG	(Pathania et al., 2014)	N/A
Genotyping primer, Deletion_F (Del^F^): TGGTAGCCCTC TTCTTGTGGA	This paper	N/A
Genotyping primer, Deletion_R (Del^R^): CTCCAAGATTC CCCCAAAAC	This paper	N/A
CYFIP1 shRNA sense:	(Yoon et al., 2014)	N/A
GATCCCCgcatgtttgtctttatgtaTTCAAGAGAtacataaagac aaacatgcTTTTTC		
CYFIP1 shRNA antisense:	(Yoon et al., 2014)	N/A
TCGAGAAAAAgcatgtttgtctttatgtaTCTCTTGAAtacataaa gacaaacatgcGGG		
Scrambled shRNA sense:	(Yoon et al., 2014)	N/A
GATCCCCttctccgaacgtgtcacgtTTCAAGAGAacgtgaca cgttcggagaaTTTTTC		
Scrambled shRNA antisense:	(Yoon et al., 2014)	N/A
TCGAGAAAAAttctccgaacgtgtcacgtTCTCTTGAAacg tgacacgttcggagaaGGG		

**Recombinant DNA**		

Gateway™ pcDNA™-DEST47 vector	Invitrogen	Cat# 12281010
pDEST-mCherry-N1	Addgene	Cat# 31907
pDEST47-humanCYFIP1^GFP^	This paper	N/A
pDEST47-human CYFIP2^GFP^	This paper	N/A
pDEST-mCherry-N1-human CYFIP1	This paper	N/A
pDEST-mCherry-N1-human CYFIP2	This paper	N/A
pcDNA6.2/V5-DEST	ThermoFisher Scientific	Cat #12489027
pDEST-V5:2A:GFP	This paper	N/A
pDEST-CYFIP1-V5:2A:GFP	This paper	N/A
pSUPER.neo+GFP	Oligoengine	Cat# VEC-pBS-0006
pEGFP-C1	Clontech	N/A
pCAG-DsRed	Addgene	Cat# 24001
GFP-actin	J. Hanley (U. of Bristol)	(Rocca et al., 2013)

**Software and Algorithms**		

Fiji/ImageJ	National Institutes of Health	https://imagej.nih.gov/ij/docs/guide/146-2.html
Metamorph	Molecular Devices	N/A
Imaris	Bitplane	N/A
GraphPad Prism	GraphPad Software	N/A
Clampfit	Molecular Devices	N/A

### Contact for Reagent and Resource Sharing

Further information and request for resources and reagents should be directed to and will be fulfilled by the Lead Contact, Josef Kittler (j.kittler@ucl.ac.uk).

### Experimental Model and Subject Details

#### Animals

All procedures for the care and treatment of animals were in accordance with the Animals (Scientific Procedures) Act 1986, and had full Home Office ethical approval. All animals were maintained under controlled conditions (temperature 20 ± 2°C; 12 h light-dark cycle). Food and water were provided *ad libitum*. Animals were group housed in conventional cages and had not been subject to previous procedures. Animals of either sex were used for all experiments. Wild-type E18 Sprague-Dawley rats were generated as a result of wild-type breeding, embryos of either sex were used for generating primary neuronal cultures.

#### Generation of the Cyfip1^NEX^ cKO mouse

The *Cyfip1* knockout (KO) mouse line (MDCK; EPD0555_2_B11; Allele: *Cyfip1*^tm2a(EUCOMM)Wtsi^) was generated using the Knockout-First strategy on C57BL/6N Taconic USA background and was obtained from the Wellcome Trust Sanger Institute as part of the International Knockout Mouse Consortium (IKMC) (Skarnes et al., 2011). The KO-first allele contains an L1L2_Bact_P cassette flanked by frt sites inserted 5′ of critical exons 4 to 6 of *Cyfip1*, disrupting gene function. The cassette can be deleted in the presence of flp recombinase by crossing with a flp-expressing strain. The flp-directed recombination produces a functional allele with the critical exons flanked by loxP sites (*Cyfip1*^F/F^) (see Figure S3 for further details). *Cyfip1* conditional knockout animals were generated by crossing *Cyfip1*^F/F^ animals with a Nex-Cre line (Goebbels et al., 2006) inducing the specific deletion of *Cyfip1* only in principal cells of the neocortex (*Cyfip1*^NEX^ cKO). P28-34 *Cyfip1*^F/F^ (control) and *Cyfip1*^NEX^ cKO of both sexes were used and were generated as a result of *Cyfip1*^F/F;NEXcre(−/−)^ x *Cyfip1*^Δ/Δ:NEXcre(+/−)^ crosses to produce littermate controls. For genotyping, DNA was extracted from ear biopsies and PCRs were performed. The *Cyfip1* WT and KO alleles were genotyped using the following three primers: (Cyfip1_234230_F (Wt^F^): 5′-TGGAAGTAATGGAACCGAACA-3′), (Cyfip1_234230_R (Wt^R^): 5′-GTAACTACCTATAATGCAGACCTGAAG-3′) and (CAS_R1_Term (Mut^R^): 5′-TCGTGGTATCGTTATGCGCC-3′). Wt^F^ and Wt^R^ produce a band from the WT allele while Wt^F^ and Mut^R^ produce a band from the KO allele. Deletion of *Cyfip1* exons 4-6 in the conditional KO allele was confirmed with primers (Deletion_F (Del^F^): 5′-TGGTAGCCCTCTTCTTGTGGA-3′) and (Deletion_R (Del^R^): 5′-CTCCAAGATTCCCCCAAAAC-3′).

#### Primary Neuronal Culture

Hippocampal neurons were obtained from E18 Sprague Dawley wild-type rat embryos of either sex as previously described (López-Doménech et al., 2016, Vaccaro et al., 2017). Briefly, embryos were placed into ice-cold Hank’s Buffered Salt Solution (HBSS) supplemented with 10 mM HEPES. Brains were isolated, meninges were removed and hippocampi were dissected. Tissue was then incubated in 0.125% trypsin diluted in HBSS + HEPES for 15 min at 37°C. Tissue was washed twice in HBSS + HEPES and triturated to a single cell suspension in attachment media (MEM supplemented with 10% horse serum, 1 mM sodium pyruvate, 0.6% glucose) using a fire-polished glass pasteur pipette. Following trituration, neurons were plated in attachment media onto pre-prepared poly-L-lysine (PLL) coated 13mm glass coverslips at a density of 35 x10^4^ cells per 6cm dish (each containing 8 coverslips). 4-6 h later media was changed to Neurobasal growth medium supplemented with B27, 1% glutaMAX and 33 mM glucose. PLL was incubated for a minimum of 3 h with coverslips at 500 μg/ml, washed twice in dH_2_0 and dried. The cultures were maintained at 37°C with 5% CO_2_. Experiments were performed at DIV 14-16 unless otherwise stated.

#### COS-7 Cell Culture

COS-7 cells were maintained in DMEM supplemented with 10% fetal bovine serum and penicillin/streptomycin (100 U/ml and 100 μg/ml respectively) at 37°C with 5% CO_2_. COS-7 cells were transfected for 48 h using the Nucleofector® device (Lonza) following the manufacturer’s protocol.

### Method Details

#### Constructs

Human CYFIP1- and CYFIP2-GFP/mCherry fusion protein constructs were generated by cloning the coding sequences into pDEST47GFP (Invitrogen) and pDEST-mCherry-N1 (Addgene, #31907) using the Gateway Cloning System (Invitrogen) and homologous recombination. For CYFIP1-2AGFP the destination vector pDEST-V5:2A:GFP was developed in house. The sequence 2A-GFP was cloned into pcDNA6.2/V5-DEST (ThermoFisher Scientific, #12489027) at the restriction enzyme site AgeI, C-terminal of the V5 sequence. In-Fusion® cloning (Takara, #638911) allowed for the 2A-GFP sequence to be in frame with V5. The CYFIP1 coding sequence was then cloned into this vector again using Gateway homologous recombination. shRNA sequences against mouse CYFIP1 and a scrambled control were cloned into pSUPER.neo+GFP (Oligoengine) following the manufacturers guidelines. Sequences were previously described and characterized by Yoon et al. (2014). For CYFIP1 shRNA, oligonucleotides (5′-GATCCCCgcatgtttgtctttatgtaTTCAAGAGAtacataaagacaaacatgcTTTTTC-3′) and (5′-TCGAGAAAAAgcatgtttgtctttatgtaTCTCTTGAAtacataaagacaaacatgcGGG-3′) were annealed and for Scrambled shRNA oligonucleotides (5′-GATCCCCttctccgaacgtgtcacgtTTCAAGAGAacgtgacacgttcggagaaTTTTTC-3′) and (5′-TCGAGAAAAAttctccgaacgtgtcacgtTCTCTTGAAacgtgacacgttcggagaaGGG-3′) were annealed. pEGFP-C1 and pCAG-DsRed were purchased from Clontech and Addgene (#24001) respectively. The GFP-actin DNA was a kind gift from J. Hanley (University of Bristol, Bristol, UK).

#### Antibodies

Primary antibodies were rabbit anti-CYFIP1 (Upstate, 07-531; ICC, 1:200; WB, 1:1000), mouse anti-GABA_A_R-β2/3 (Neuromab, MAB341; WB, 1:500), guinea pig anti-GABA_A_R-γ2 (Synaptic Systems, 224 004; ICC, 1:500), mouse anti-gephyrin (Synaptic Systems, 147 011; ICC, 1:500; IHC, 1:500; WB, 1:500), rat anti-GFP (Nacalai-Tesque, GF090R; ICC, 1:1000), mouse anti-GFP (Neuromab, 73-131; WB, 1:100), mouse anti-GIT1 (Neuromab, N39/B8; WB, 1:500), rabbit anti-Homer (Synaptic Systems, 160 002; ICC, 1:500; WB, 1:500), rabbit anti-neuroligin 2 (Synaptic Systems, 129 202; WB, 1:1000), mouse anti-neuroligin 3 (Neuromab, N110/29; WB, 1:100), mouse anti-PSD95 (Neuromab, K28/43; ICC, 1:500; WB, 1:1000), rabbit anti-β-PIX (Upstate, 07-1450; WB, 1:2000), mouse anti-β-tubulin (Sigma, T5293; WB, 1:1000), rabbit anti-vGAT (Synaptic Systems, 131 003; ICC, 1:1000), guinea pig anti-vGLUT (Synaptic Systems, 135304; ICC, 1:1000), mouse anti-V5 (Invitrogen, R960-25, ICC, 1:1000; WB, 1:1000). Secondary fluorescent antibodies were conjugated to Alexa Fluor 488, 568, 647 (1:1000, Molecular Probes). Anti-rabbit and anti-mouse HRP conjugated secondaries were from Jackson ImmunoResearch (WB, 1;1000).

#### Primary Neuronal Transfections

Hippocampal neurons were transfected using Lipofectamine-2000 (ThermoFisher Scientific). For two 13mm coverslips in individual wells of a 24 well plate, 1 μg of DNA was combined with 100 μl of unsupplemented Neurobasal growth medium (NB) in one tube and 2 μl Lipofectamine with 100 μl NB in another tube. Following 5 min incubation at room temperature the Lipofectamine solution was gently combined with the DNA and incubated for 20 min at room temperature to complex. 300 μl of prewarmed NB + 0.6% glucose was added to the complex solution and gently mixed. Conditioned media was removed from coverslips and kept, 250 μL of the complex solution was then dropped carefully onto each coverslip. Coverslips were incubated at 37°C for 2 h followed by replacing the transfection solution with 1ml pre-warmed conditioned media. Hippocampal neurons were transfected 4-5 days prior to use.

#### Immunocytochemistry and Immunohistochemistry

Hippocampal cultures were fixed in 4% PFA (PBS, 4% paraformaldehdye, 4% sucrose, pH 7) for 7 min then permeablised for 10 min in block solution (PBS, 10% horse serum, 0.5% BSA, 0.2% Triton X-100). Coverslips were incubated with primary antibody diluted in block solution for 1 h, washed in PBS then incubated for 1 h with secondary antibody. Finally coverslips were washed and mounted onto glass slides using ProLong Gold antifade reagent (Invitrogen). For surface labeling, block solution was used without detergent.

For immunohistochemistry, adult mouse brains of either sex were fixed in 4% PFA overnight and cryoprotected in 30% sucrose/PBS solution overnight before freezing at −80°C. The brain samples were embedded in tissue freezing compound (TissueTek OCT) and 30 μm brain sections were generated using a Cryostat (Bright Instruments, Luton, UK). Free floating thin sections were permeablised for 4-5 h in block solution (PBS, 10% horse serum, 0.5% BSA, 0.5% Triton X-100, 0.2 M glycine) then incubated with primary antibody diluted in block solution overnight at 4°C. For mouse primary antibodies, slices were first incubated overnight at 4°C with mouse Fab fragment (1:50 with block solution; 115-007-003, Jackson ImmunoResearch) to reduce background staining on the mouse tissue. Slices were washed 4-5 times in PBS for 2 h then incubated for 3-4 h with secondary antibody at room temperature. The slices were then washed 4-5 times in PBS for 2 h and mounted onto glass slides using Mowiol mounting medium. For antigen retrieval, slices were incubated in sodium citrate solution at 80°C for 40 mins and then washed 3x in PBS prior to blocking.

#### Confocal Microscopy and Image Analysis

All confocal images were acquired on a Zeiss LSM700 upright confocal microscope using a 63X oil objective (NA: 1.4) unless otherwise stated. For synaptic localization, enrichment and cluster analysis experiments from cultured neurons, a single plane image of each cell was captured using a 0.5X zoom. From this, 3 sections of primary or secondary dendrite, ∼100 μm from the soma, were imaged with a 3.5X zoom (equating to a 30 μm length of dendrite). For brain sections from adult male and female fixed brains, 2 low- magnification regions of the hippocampus were captured using a 63X objective and 0.5X zoom. From this, 3 regions were imaged within each hippocampal strata with a 2X zoom for analysis. Acquisition settings and laser power were kept constant within all experiments.

Line scans used for protein localization were performed in ImageJ using the PlotProfile function (NIH, Bethesda, MD, USA), pixel intensity was calculated as a function of distance along a manually drawn line and plotted on a graph. Synaptic enrichment and cluster analysis was carried out using Metamorph (Molecular Devices, Sunnyvale, CA, USA). Analysis was carried out on the zoom dendrite images and then averaged to give a value per cell. To quantify protein enrichment at synaptic sites, the protein fluorescence intensity was measured as the average intensity within the labeled synaptic puncta and normalized to the average intensity of the total process. For synaptic cluster analysis, the length of dendrite was traced to generate a dendritic region of interest (ROI). This ROI was transferred to all cluster channels. A user-defined threshold was then applied to each synaptic marker channel and regions were generated around the thresholded area within the dendrite ROI. Number of regions and total area of regions per 30 μm of dendrite were quantified as a readout for synaptic clusters. Clusters smaller than 0.01 μm^2^ were excluded from the number of regions analysis. Thresholds were set individually for each cluster channel and kept constant across treatment conditions within an experiment. For brain sections labeled with antibodies against gephyrin and VGAT, the Synapse Counter plugin for ImageJ (NIH, Bethesda, MD, USA) was used. Background subtraction and max filter parameters were set to 10 and 1 respectively. Clusters greater than 0.095 μm^2^ and less than 1 μm^2^ were considered for total cluster area analysis. For spine morphology analysis of cultured neurons, confocal image stacks were acquired. Spines were manually identified on 100-200 μm long dendritic filaments and analyzed in Imaris software (Bitplane, Zurich, Switzerland). For spine subtype classification custom parameters were used. Classification was entirely automated until the final step where blatant errors in classification were removed.

Time-gated STED imaging was carried out on a Leica TCS SP8 STED 3x microscope running LAS X (Version 2.01.14392) acquisition software using a 100x HC PL APO CS2 oil immersion objective (NA 1.4). Oregon Green 488 (Thermo Fisher Scientific) and Abberoir Star 440SX (Sigma) were excited using the 488nm line (15%) and the 405nm output (20%) from a white light laser (WLL, operating at 70% of its nominal power) respectively. Fluorescence depletion, and therefore super-resolution, was accomplished using a 592nm STED laser (1.5W nominal power, 40% for both Oregon Green 488 and Abberoir Star 440SX). All 2048 × 2048 pixel single plane images were acquired at a scan speed of 400 Hz in bidirectional scan mode. The pixel size of 30.4nm^2^ was optimized for STED imaging. The fluorescence signal was then detected by a Hybrid Detector (HyD, Standard mode) after passing through an Acousto-Optical Beam Splitter (AOBS, detection range 482 – 510nm for Abberoir Star 440SX and 520 – 565nm for Oregon Green 488 when doing two-color imaging). Time-gated detection was turned on to further improve the resolution in the STED images (0.5 – 6.0ns). The detector gain was adjusted so that no saturation occurred in the images. All 2D STED images were deconvolved using the CMLE (Classic Maximum Likelihood Estimation) algorithm in SVI Huygens Professional (Version 15.10.1p2) to improve the signal-to-noise ratio.

#### Proximity Ligation Assay

Proximity ligation assays (Duolink®) were carried out using anti-CYFIP1 and anti-gephyrin antibodies or anti-gephyrin alone for control proximity ligation assays. Neurons were fixed and blocked as for immunofluorescence and incubated with primary antibodies. Following primary antibody incubation, cells were washed in PBS before incubation with secondary antibodies conjugated with oligonucleotides. Ligation and amplification reactions were conducted at 37°C, as described in the Duolink® manual, before mounting and visualization using confocal microscopy (Norkett et al., 2016). For PLA analysis, confocal image stacks with a X0.5 zoom and voxel dimensions 0.39 μm x 0.39 μm x 0.57 μm were acquired. Analysis was carried out on maximum projection images using Metamorph (Molecular Devices, Sunnyvale, CA, USA). A user-defined threshold was applied to each image which best detected PLA puncta and kept constant within an experiment. Puncta were then counted per field of view.

#### Electrophysiology in Dissociated Cultures

Whole-cell recordings were performed on transfected cultured hippocampal neurons at 14-17 DIV. Neurons were held at −70 mV. Patch electrodes (4-5 MΩ) were filled with an internal solution containing (in mM): 120 CsCl, 5 QX314 Br, 8 NaCl, 0.2 MgCl_2_, 10 HEPES, 2 EGTA, 2 MgATP and 0.3 Na3GTP. The osmolarity and pH were adjusted to 300 mOsm/L and 7.2 respectively. The external artificial cerebro-spinal fluid (ACSF) solution consisted of the following (in mM): 125 NaCl, 25 NaHCO_3_, 2.5 KCl, 2 MgCl_2_, 1.25 NaH_2_PO_4_, 2 CaCl_2_, and 25 glucose saturated with 95% O_2_/5% CO_2_ (pH 7.4, 320 mOsm). This solution was supplemented with NBQX (20 μM), APV (50 μM) and TTX (1 μM) to isolate mIPSCs or with bicuculline (20 μM) and TTX (1 μM) for mEPSCs recording. All recordings were performed at room temperature (22-25°C). The access resistance, monitored throughout the experiments, was < 20 MΩ and results were discarded if it changed by more than 20%. Miniature events and theirs kinetics were analyzed using template-based event detection in Clampfit (Molecular Devices, Sunnyvale, CA, USA). Total charge transfer was calculated as described by Peden et al. (2008). For all electrophysiological experiments, the experimenter was blind to the condition/genotype of the sample analyzed.

#### Acute Hippocampal Slice Electrophysiology

To prepare acute hippocampal slices, male and female mice aged postnatal day 28-34 were used. Immediately after decapitation, the brain was removed and kept in ice-cold dissecting solution. Transverse hippocampal slices (300 μm) were obtained using a vibratome (Leica, VT–1200S). Slices were stored at 35°C for 30 min after slicing and then at 22°C. For the dissection and storage of slices, the solution contained (in mM): 87 NaCl, 25 NaHCO_3_, 10 glucose, 75 sucrose, 2.5 KCl, 1.25 NaH_2_PO_4_, 0.5 CaCl_2_ and 7 MgCl_2_ saturated with 95% O_2_/5% CO_2_. For patch-clamp experiments, CA1 pyramidal neurons were identified under infrared-differential interference contrast (DIC) imaging with a water-immersion 60X objective (Olympus) and whole-cell recordings were performed as described above for cultured cells.

#### Preparation of Brain Lysates

Adult WT and conditional KO male or female whole brains or cortical regions were sonicated in lysis buffer (50 mM HEPES pH 7.5, 0.5% Triton X-100, 150 mM NaCl, 1 mM EDTA, 1 mM PMSF in the presence of antipain, pepstatin and leupeptin) then left to rotate at 4°C for 1 h. Membranes were pelleted by centrifugation at 14000 g for 15 min at 4°C. Protein content of the supernatant was assayed by BioRad protein assay. Samples were then suspended in 3X protein sample buffer and analyzed by SDS-PAGE and western blotting. Briefly, protein samples were separated by SDS-PAGE on 10% Tris-Glycine gels and blotted onto nitrocellulose membranes (GE Healthcare Bio-Sciences). Membranes were blocked for 1 h in milk (PBS, 0.05% Tween, 4% milk), incubated in primary antibodies diluted with milk overnight at 4°C before incubation in an appropriate HRP-conjugated secondary antibody for 1 h at room temperature. The blots were developed with an ECL-Plus detection reagent (GE Healthcare Bio-Sciences). Densitometric analysis was performed in ImageJ (NIH).

#### Golgi Staining

Dendritic and spine morphology in P30 mice was analyzed using the FD Rapid Golgi Stain kit (FD NeuroTechnologies, Baltimore, MD, USA) and Neurolucida (MBF Bioscience, Williston, VT, USA). Golgi-impregnated brains were sliced at 100 μm using a vibratome (Leica Microsystems, Heerbrugg, Switzerland). Well-isolated hippocampal CA1 neurons were imaged at 20X using the Neurolucida software system and an upright light microscope with a motorized stage (MBF Bioscience). The entire dendritic tree (apical and basal) was traced and reconstructed. 3-dimensional Sholl analysis of reconstructions was performed using a custom MATLAB script. For spine analysis, 50 μm z stacks of 2 μm step size were imaged at 40X using a ZEISS Axio Scan system and sections of basal dendrite were randomly selected for analysis. Spine length and head width were manually traced in ImageJ and the data analyzed using a custom Excel macro.

### Quantification and Statistical Analysis

All data were obtained using cells from at least three independent primary culture preparations or at least three independent animals per genotype. Repeats for experiments and statistical tests carried out are given in the figure legends as N numbers and refer to number of cells unless otherwise stated. All statistical analysis was carried out using GraphPad Prism (GraphPad Software, CA, USA) or Microsoft Excel. Data were tested for normal distribution with D’Agostino and Person to determine the use of parametric (unpaired Student’s t test, one-way ANOVA, two-way ANOVA) or non-parametric (Mann-Whitney, Kruskal-Wallis) tests. Appropriate post hoc tests were carried out in analyses with multiple comparisons and are stated in figure legends. p values < 0.05 were considered significant. Data are shown as mean ± standard error of the mean (SEM).

## Supplemental Information

Document S1. Figures S1–S4

Document S2. Article plus Supplemental Information
