# Supplementary material for: Autism and Schizophrenia-Associated CYFIP1 Regulates the Balance of Synaptic Excitation and Inhibition
Source: Cell Rep. 2019 Feb 19;26(8):2037–2051.e6. doi: 10.1016/j.celrep.2019.01.092 (PMC6381785; doi:10.1016/j.celrep.2019.01.092)
Supplement: Document S1. Figures S1–S4 [file mmc1.pdf]

Cell Reports, Volume *26*

Supplemental Information

Autism and Schizophrenia-Associated CYFIP1 Regulates the Balance of Synaptic Excitation and Inhibition

Elizabeth C. Davenport, Blanka R. Szulc, James Drew, James Taylor, Toby Morgan, Nathalie F. Higgs, Guillermo López-Doménech, and Josef T. Kittler

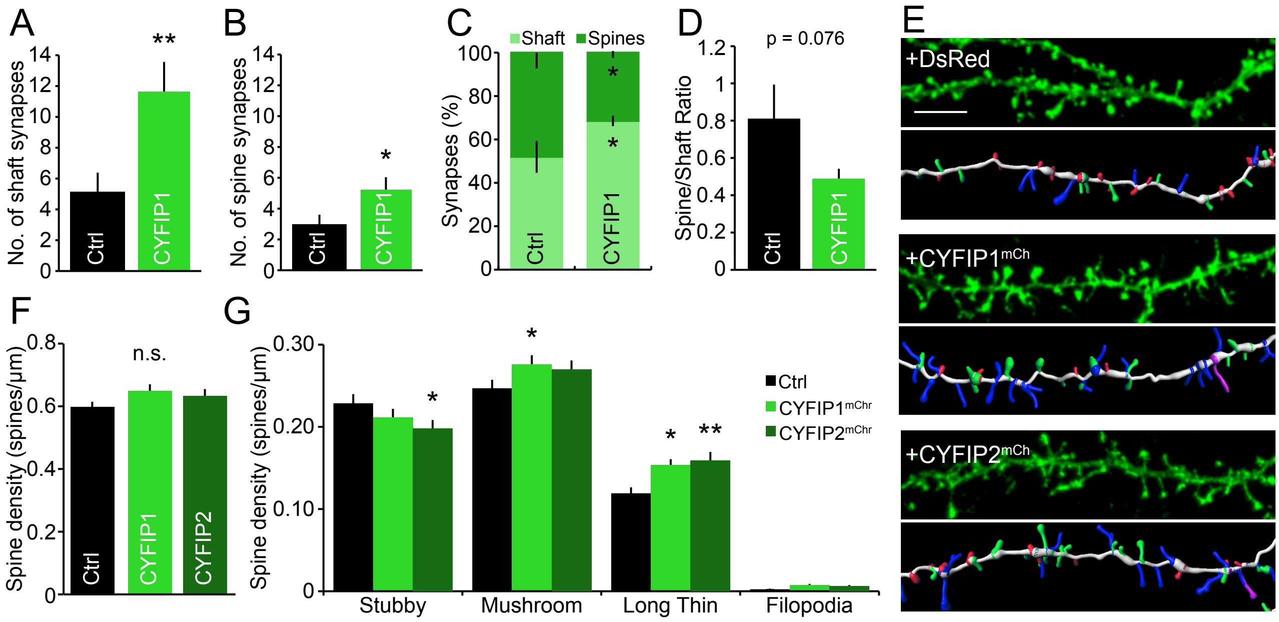

Figure S1: CYFIP1 overexpression redistributes excitatory synapses between dendritic spines and shaft, impacts spine morphology but does not alter spine density.
Related to Figure 3.

(A-B) $CYFIP1^{GFP}$ and GFP control overexpressing neurons were fixed and stained at DIV14 with antibodies to the pre and post excitatory synaptic markers VGLUT and PSD95. Number of synapses, considered as PSD95 and VGLUT positive clusters, were quantified within (A) the dendritic shaft and (B) dendritic spines. $CYFIP1^{GFP}$ overexpression resulted in a significant increase in number of synapses on both the shaft and spines (shaft synapses: from 5.1 ± 1.2 to 11.6 ± 1.9 for CYFIP1, p = 0.0084; spine synapses: from 2.9 ± 0.6 to 5.2 ± 0.8 for CYFIP1, p = 0.039; both n = 15-16 cells from 3 independent preparations; Student's t-test).
(C) Graph representing the proportion of excitatory synapses located on dendritic spines compared to the dendritic shaft (shaft synapses: from 52 ± 7.3% to 68.4 ± 2.4% for CYFIP1, n = 14 cells from 3 independent preparations, p = 0.036, Student's t-test).
(D) The ratio of spine to shaft excitatory synapses in control compared to $CYFIP1^{GFP}$ overexpressing cells (from 0.8 ± 0.2 to 0.5 ± 0.1, n = 14 cells from 3 independent preparations, p = 0.076 n.s., Student's t-test).
(E) Mature hippocampal neurons were transfected for 4 days with $actin^{GFP}$ to label cell morphology and DsRed control, $CYFIP1^{mCherry}$ or $CYFIP2^{mCherry}$, fixed and imaged (upper panels: representative images; lower panels: 3D reconstruction). Colour key for spine 3D reconstruction: green = mushroom, red = stubby, blue = long and thin, pink = filopodia. Scale bar, 5 μm.
(F) Dendritic spine analysis revealed no change in total spine density (from 0.6 ± 0.02 to 0.65 ± 0.02 for CYFIP1 and 0.63 ± 0.02 for CYFIP2; n=49-66 filaments per condition; 1-way ANOVA, Dunn's post-hoc multiple comparison, n.s.).
(G) Quantification of spine subtype density in $CYFIP1^{mCherry}$ and $CYFIP2^{mCherry}$ overexpressing cells (Spines/μm, stubby: from 0.23 ± 0.01 to 0.21 ± 0.01 for CYFIP1 and 0.20 ± 0.01 for CYFIP2; mushroom: from 0.25 ± 0.01 to 0.28 ± 0.01 for CYFIP1 and 0.27 ± 0.01 for CYFIP2; long,thin: from 0.12 ± 0.01 to 0.15 ± 0.01 for CYFIP1 and 0.16 ± 0.01 for CYFIP2; filopodia: from 0.002 ± 0.001 to 0.007 ± 0.002 for CYFIP1 and 0.006 ± 0.001 for CYFIP2; n = 49-66 filaments per condition; 2-way ANOVA, Tukey's post-hoc multiple comparison).
*p < 0.05, **p < 0.01.
Bars indicate mean and error bars s.e.m.

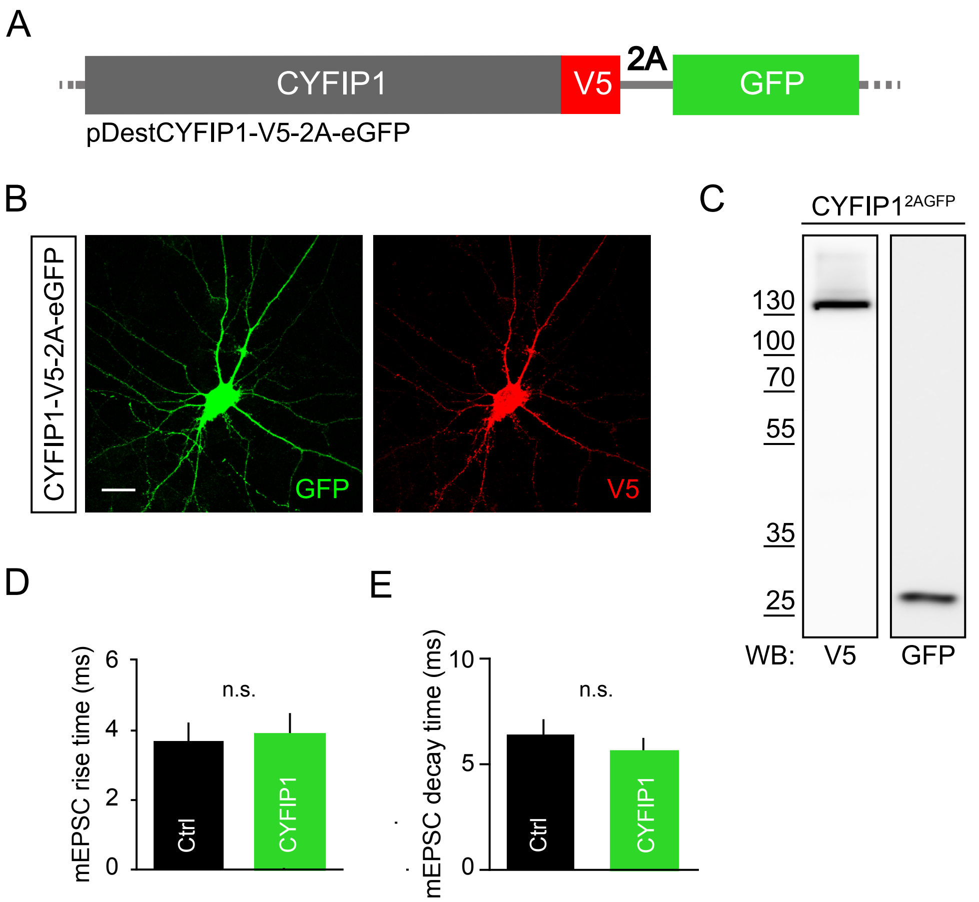

Figure S2: Development and application of the CYFIP1^{2AGFP} construct. Related to Figure 4.

To facilitate live cell identification of CYFIP1 overexpressing cells for electrophysiological analysis, CYFIP1 was dually expressed with GFP from a plasmid by the addition of a 2A sequence between the CYFIP1 and GFP cDNA.
(A) Schematic showing CYFIP1 in the 2A vector. Expression of this vector produces CYFIP1 fused to the small V5 tag and independent expression of cytosolic GFP. Expression of CYFIP1 and GFP is under the same CMV promoter. The 2A sequence in the mRNA causes the transcribing ribosome to skip resulting in the termination of the initial transcribed CYFIP1 sequence and the formation of a new polypeptide at the start of the GFP sequence.
(B) Hippocampal cells transfected with CYFIP1^{2AGFP}. Cells were labelled with antibodies to V5 to confirm CYFIP1 expression and GFP to amplify the cytosolic GFP expression. Scale bar, 20 µm.
(C) Western blot of COS7 cell lysate sample transfected with CYFIP1^{2AGFP} and probed with antibodies to V5 and GFP. Bands are detected at the expected weight for GFP alone and CYFIP1^{V5} indicating that ribosome skipping and protein expression is occurring correctly.
(D,E) mEPSCs were recorded from DIV14-16 neurons overexpressing CYFIP1^{2AGFP} or GFP. Calculation of rise (D) and decay (E) time kinetics from these recordings showed no change between GFP and CYFIP1 overexpressing cells (rise time: from 3.7 ± 0.5 ms to 3.9 ± 0.6 ms; n = 12 cells from 3 preparations; p = 0.701 n.s.; Mann-Whitney; decay time: from 6.4 ± 0.7 ms to 5.6 ± 0.6 ms; n = 12 cells from 3 preparations; p = 0.387 n.s.; Student's t-test).

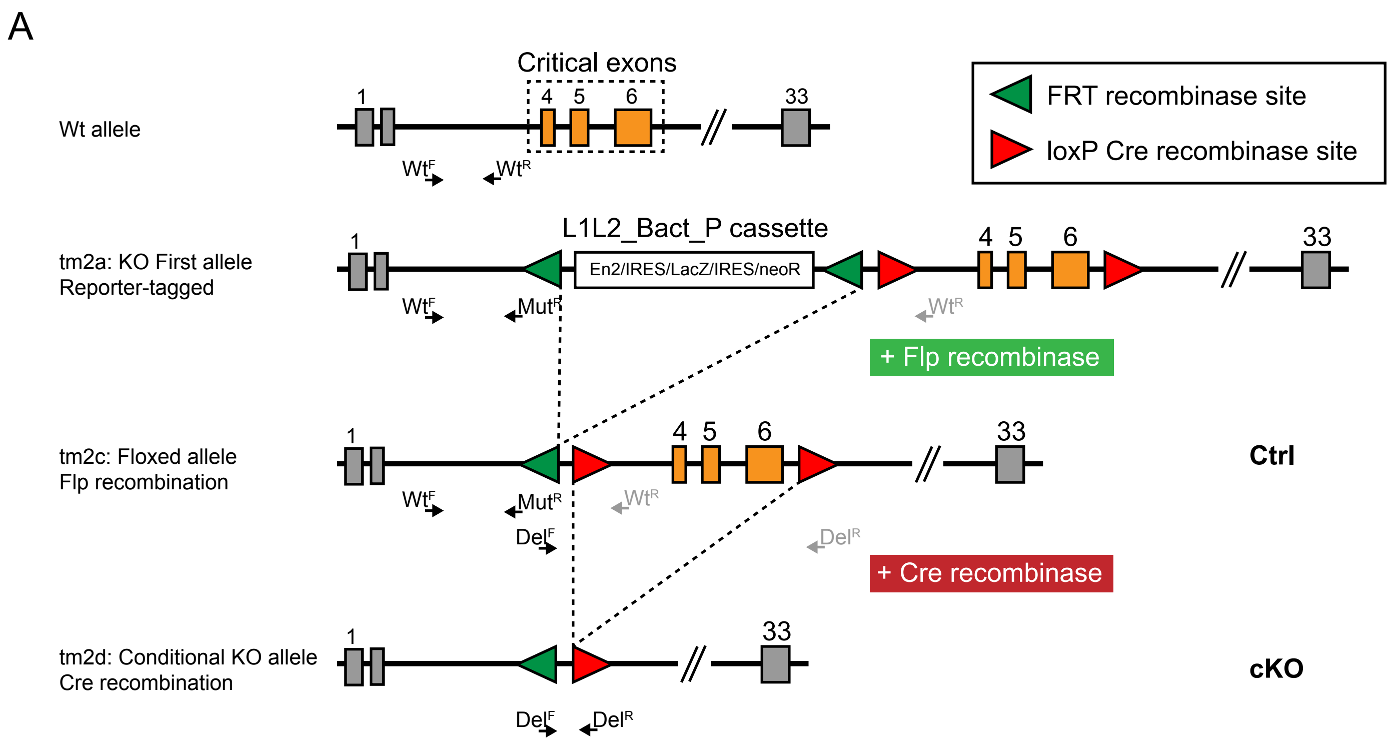

Figure S3: Development and characterisation of the CYFIP1 conditional knockout mouse. Related to Figure 5.

(A) A schematic of the knockout (KO)-first allele system, demonstrating the generation of the *Cyfip1* floxed allele (tm2c) following Flp recombination of the KO-first allele (tm2a) and then finally the formation of the conditional KO (cKO) allele (tm2d) following Cre recombination of the floxed allele. The KO-first allele contains an IRES:lacZ trapping cassette and a promoter-driven neo cassette inserted 5' of critical exons 4 to 6 of *Cyfip1*, disrupting gene function. The cassettes are bound by two frt sites (green triangles) and are extruded in the presence of Flp recombinase, generating the floxed allele. The critical exons are flanked by loxP sites (red triangles), by subjecting the floxed allele to Cre recombination the cKO allele is achieved (Skarnes et al., 2011).

Primer pair WT^F and WT^R produce a PCR product of 259 base pairs (bp) from the WT allele, these primers are too distant from each other to produce a product from the KO-first allele and produce a shifted 'ghost band' from the floxed allele. Primers WT^F and Mut^R produced a 182 bp product from the KO-first allele with Mut^R annealing at the very 5' region of the LacZ cassette. Primers Del^F and Del^R produced a 499 bp product from the cKO allele but are too distant from each other to produce a product from the floxed allele.

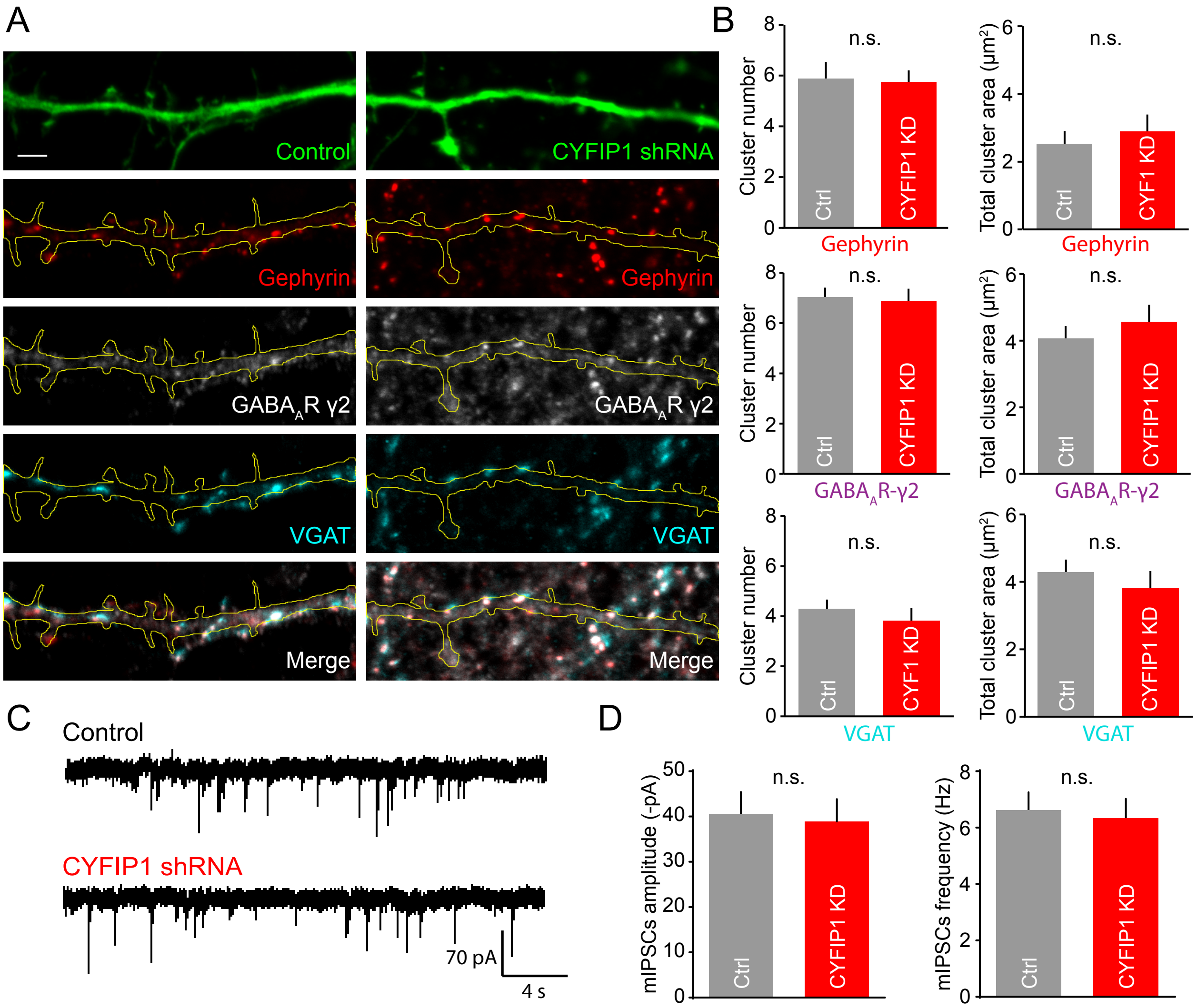

Figure S4: The effect of CYFIP1 acute knock-down on inhibitory synaptic structure and transmission.
Related to Figure 7.

(A) Representative confocal images of mouse hippocampal neurons transfected with CYFIP1 shRNA or scrambled control shRNA for 4-5 days before fixing at DIV14 and labelling with antibodies to gephyrin, surface GABA$_A$R-γ2 subunit and VGAT. Scale bar, 2 μm.
(B) Synaptic cluster analysis showing no change in either gephyrin, surface GABA$_A$R-γ2 subunit or VGAT cluster number or total immunolabelled area between CYFIP1 shRNA knock-down (KD) and scrambled control neurons (Gephyrin: cluster number: from 5.9 ± 0.7 to 5.7 ± 0.5; cluster area: from 2.5 ± 0.4 μm^2 to 2.9 ± 0.5 μm^2; GABA$_A$R-γ2: cluster number: from 7.0 ± 0.6 to 6.9 ± 0.9; cluster area: from 4.1 ± 0.6 μm^2 to 4.6 ± 0.9 μm^2; VGAT: cluster number: from 4.3 ± 0.4 to 3.8 ± 0.4; cluster area: from 1.9 ± 0.2 μm^2 to 1.8 ± 0.3 μm^2; n = 21 cells from 3 preparations; Student's t-test; ns).
(C) Representative traces of miniature inhibitory postsynaptic currents (mIPSCs) recorded from control scrambled shRNA and CYFIP1 shRNA expressing cultured hippocampal neurons at DIV14-16.
(D) Pooled data of mIPSCs showing no change in mean mIPSC amplitude or frequency between control and CYFIP1 shRNA knock-down (KD) neurons (mIPSC amplitude: from 40.6 ± 4.9 -pA to 38.9 ± 5.0 -pA, p = 0.8136 n.s.; frequency: from 6.6 ± 0.6 Hz to 6.3 ± 0.7 Hz, p = 0.7575 n.s.; all n = 10 cells from 2 preparations; Student's t-test).
